# Supplementary material for: Parallel Genome-wide Profiling of Coding and Non-coding RNAs to Identify Novel Regulatory Elements in Embryonic and Maturated Heart
Source: Mol Ther Nucleic Acids. 2018 May 4;12:158–73. doi: 10.1016/j.omtn.2018.04.018 (PMC6023836; doi:10.1016/j.omtn.2018.04.018)
Supplement: Document S1. Supplemental Text and Figures S1–S6 [file mmc1.pdf]

OMTN, Volume *12*

Supplemental Information

Parallel Genome-wide Profiling of Coding and Non-coding RNAs to Identify Novel Regulatory Elements in Embryonic and Maturated Heart

Davood Sabour, Rui S.R. Machado, José P. Pinto, Susan Rohani, Raja G.A. Sahito, Jürgen Hescheler, Matthias E. Futschik, and Agapios Sachinidis

Supplementary Figures

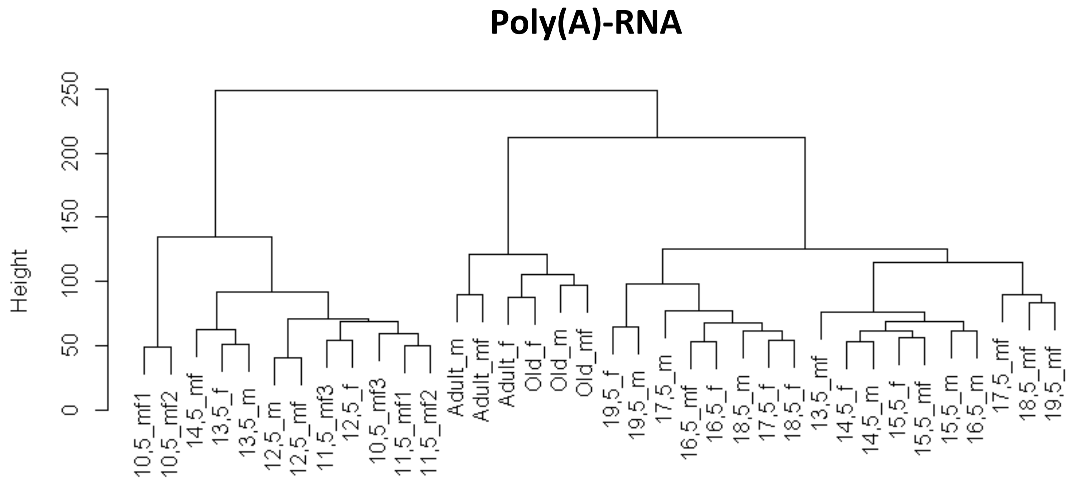

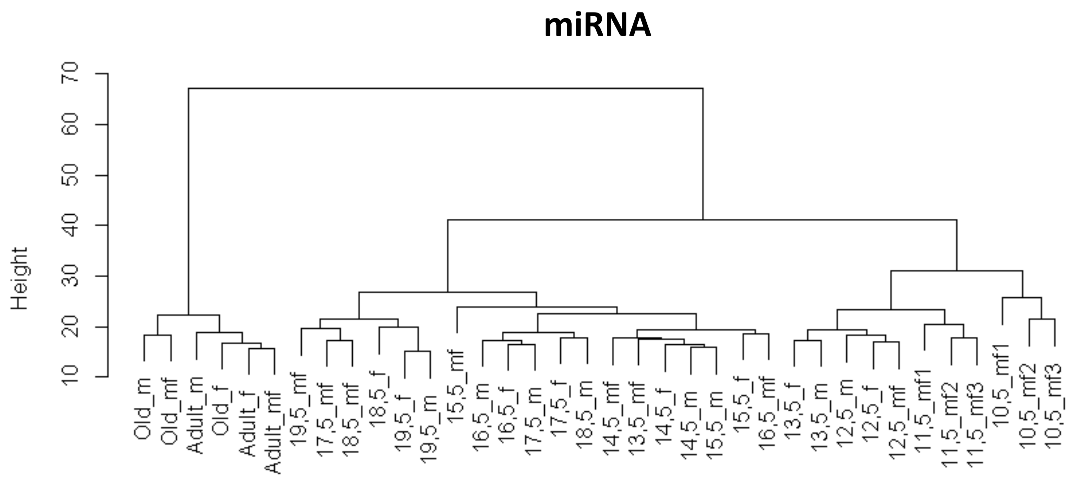

Figure S1. **Clustering dendrograms** produced based on both full poly(A)-RNA and miRNA microarray profiles.

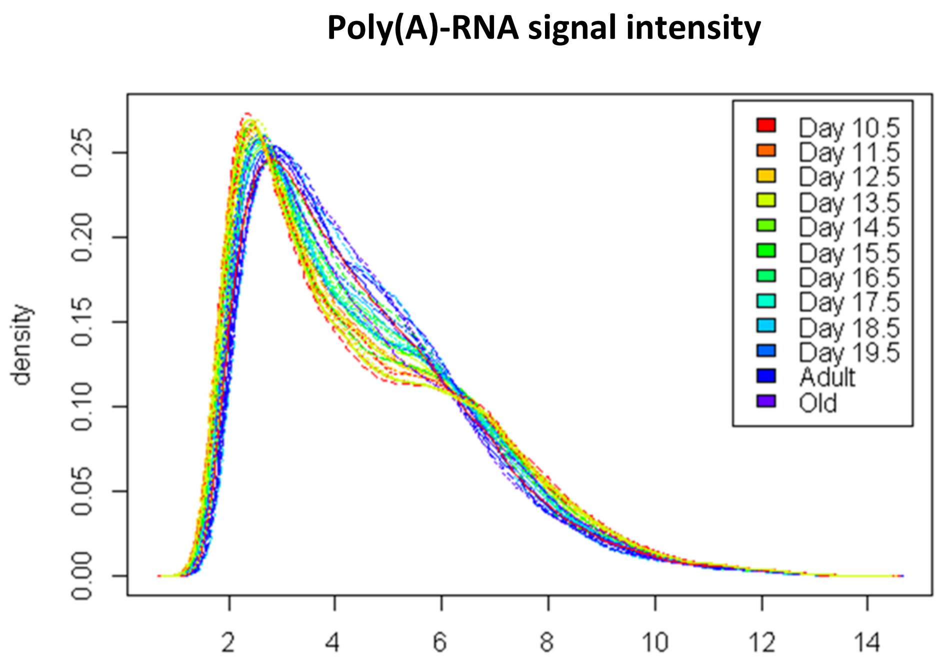

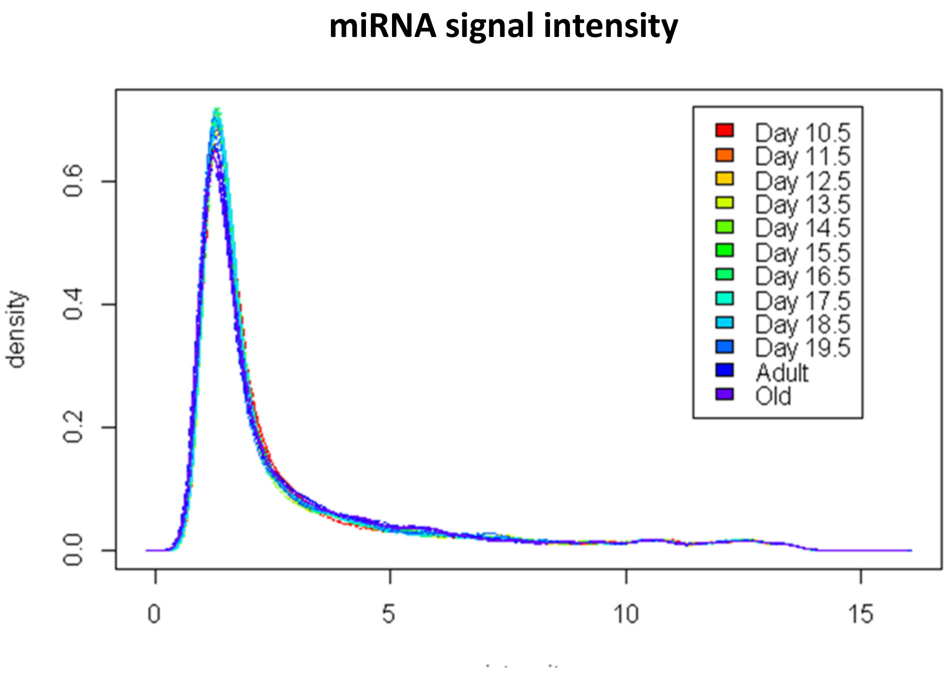

Figure S2. **Density plots of log2-tranformed expression intensities**. The distribution for poly(A)-RNA displays a notable shoulder for logged expression values around 6 at earlier developmental stages (E11.5, E12.5, E13.5).

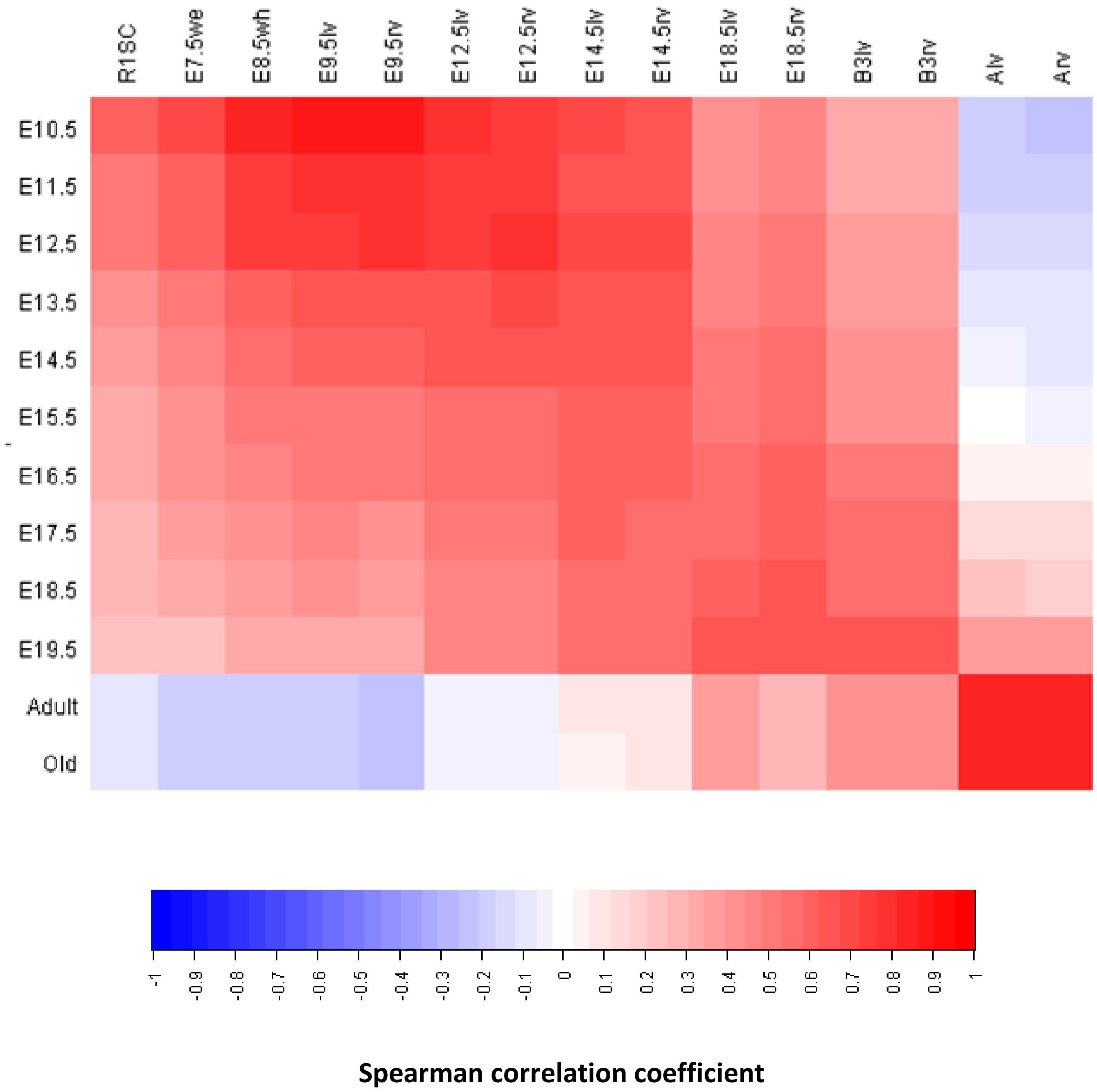

Figure S3. Heatmap of correlation coefficients derived by cross-correlation with alternative microarray study (Li et al., 2014). Color represent Spearman correlation coefficient between expression profiles from our study and the study by Li *et. al*. The bar below the heatmap displays the color scheme: red indicates positive correlation while blue indicates negative correlation (or anti-correlation). In the labels for the time series by Li *et. al.* "we" stands for "whole embryo", "wh" for "whole heart", "lv" for "left ventricle", "rv" for "right ventricle", and "B" day after birth. Moreover, this dataset included an expression profile of an undifferentiated murine embryonic stem cell line (R1SC).

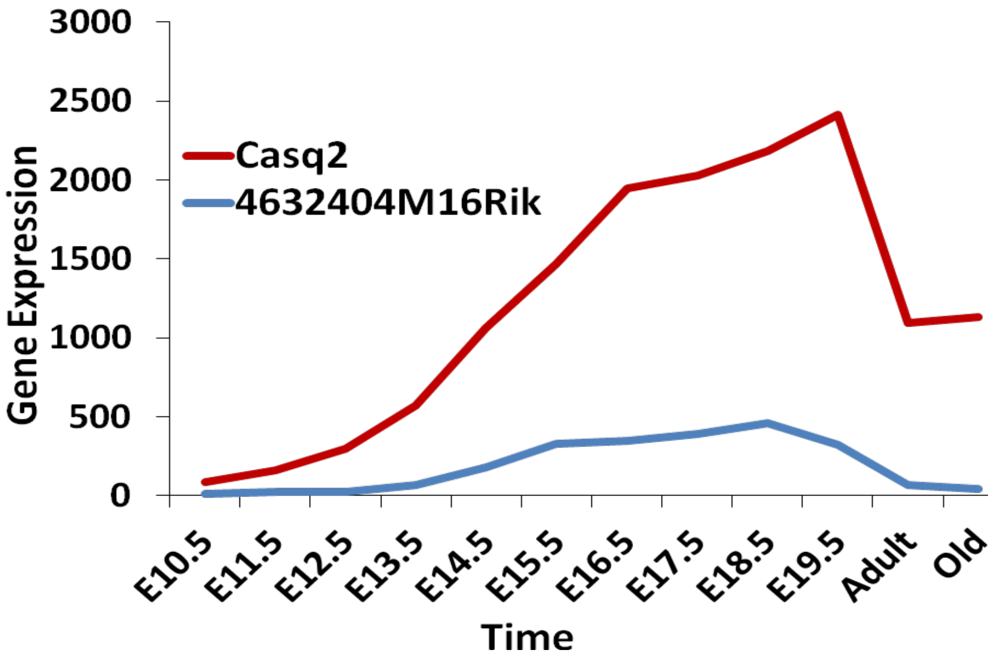

Figure S4: Expression profiles of Casq2 and the intronic lncRNA 4632404M16Rik. After reaching a peak at day 18.5, the expression of the lncRNA drops by a factor of ten in old tissue.

A

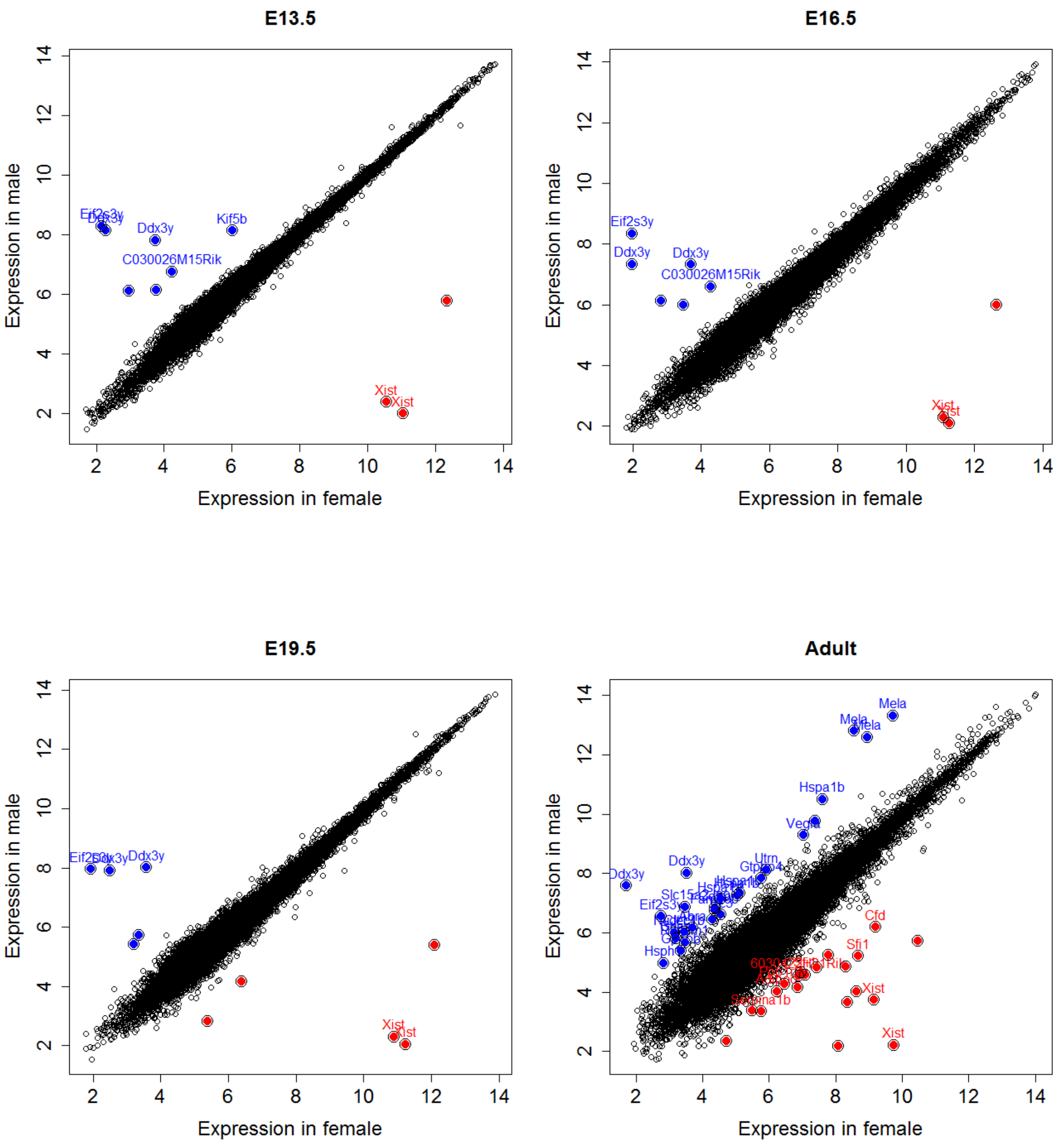
E13.5
E16.5
E19.5
Adult
Expression in male
Expression in female
Eif2s3y
Ddx3y
Kif5b
C030026M15Rik
Xist
Mela
Hspa1b
Utrn
Cfd
Sfi1

B

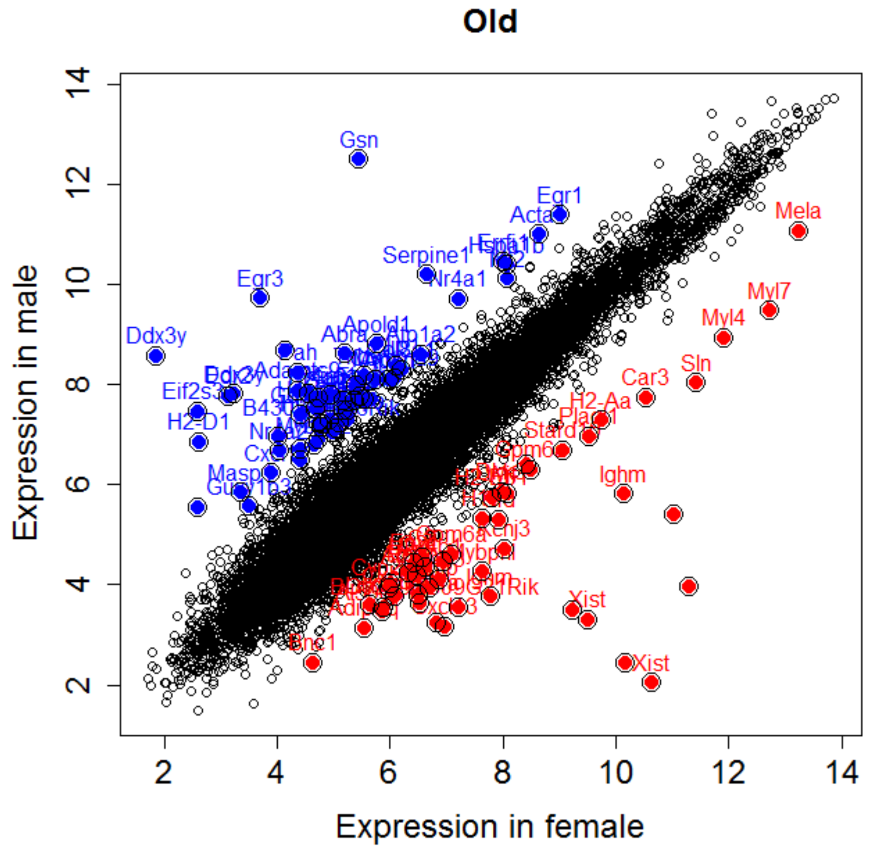

C

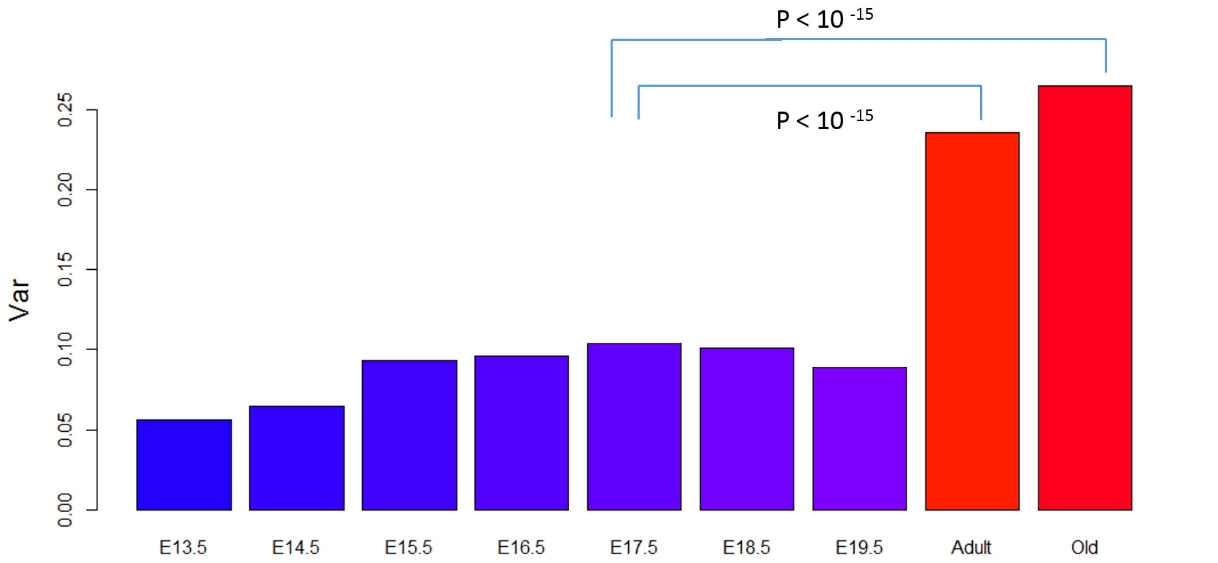

Figure S5. Comparison of gene expression in male and female samples. A) Scatterplots of logged signal intensities for male vs female samples of E13.5, E16.5, E19.5 and young adult mice. Red dots mark probe sets with signal intensities that are more than 4-fold larger in female than in male samples. Blue dots indicate probe sets with at least 4-fold larger expression in male samples. **B)** Scatterplot for old heart tissue. **C)** Variance of differential expression between male and female samples for different time points. P-values were derived using the F-test.

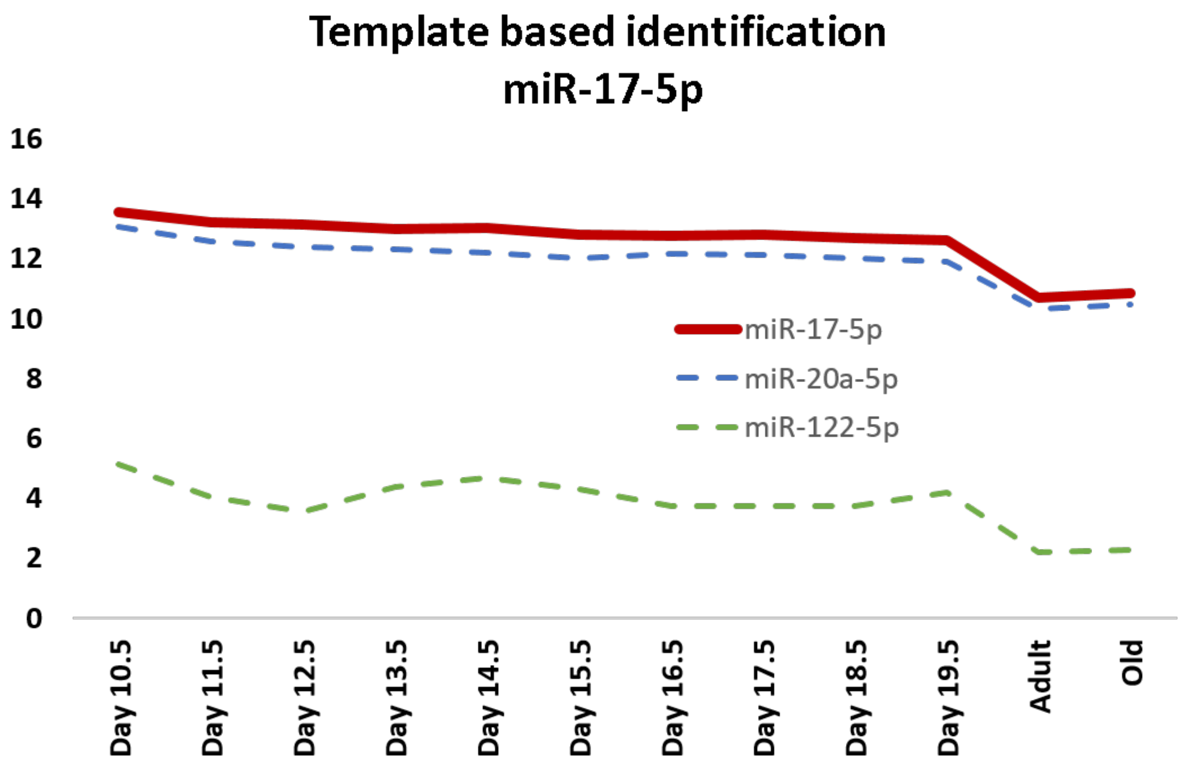

Figure S6. Example for detection of miRNAs with similar profiles. Here we used mir-17-5p as template and searched for miRNA with similar expression patterns in our miR transcriptome data set.

Supplementary Text

a. Comparison with previous microarray study of murine heart development

Recently, a similar study poly(A)-RNA abundance during the murine heart development was performed using the same type of the Affymetrix microarrays as we used in our study (Mouse Genome 430 2.0 arrays).[1] To assess the reproducibility of our study, we compared the expression values detected by the two studies.

For this purpose, we calculate the cross-correlation for the expression profiles captured at the different heart developmental stages. Such approach avoids the necessity that both studies include the same time points, which is not the case here. The alternative study of Li and co-workers[1] had lower temporal resolution (i.e. no measurements were taken for E10.5, E11.5, E13.5, E16.5, E17.5, E19.5), and started earlier during development making a direct alignment challenging. Furthermore, it included distinct tissue samples for left and right ventricle. Nevertheless, we would expect a high pair-wise correlation of similar time points using the cross-correlation approach if expression profiles of both studies agree. The expression profiles were defined here as the set of intensity values (averaged over replicates) of the 3540 Affymetrix probe sets that showed differential expression in our study. The microarray data by Li et al (2014)[1] were downloaded from GEO and processed in the same manner as ours. The results of the cross-correlation were displayed as heatmap, in which red colour represents correlation and the blue colour represents anti-correlation (**Figure S3**).

The comparison showed that the expression profiles for similar time points are highly correlated between the two studies. For example, a correlation of 0.87 was obtained for the expression values of E10.5 in our study and E9.5 of the alternative study. Inspection also reveals that both time series agree in their relative temporal alignment. The level of correlation decreases with increased distance in time. For instance, the measurements for adult tissues in both studies are strongly correlated, while anti-correlated with early developmental time points of the other study. Based on the obtained results we conclude that detected gene expression intensities in our study are consistent with those previously reported.[1]

b. Implementation of HeartmiR tool

HeartmiR is based on the integration of a large number of potential miRNAs-mRNAs interactions derived from different sources with the expression data that we obtained by parallel genome-wide profiling of mRNA and miRNA undertaken in our study. To obtain a comprehensive set of miRNA-mRNA interactions, we integrated five different datasets of miRNA-mRNA interactions that are publically available. These interactions are based on experiments[2] or computational predictions.[3-6] In order to obtain interactions of high confidence, each dataset was individually examined and filtered following the recommendations of the creators of the resources:

- **microRNA.org** (www.microrna.org): The two files labelled with “Good mirSVR score, Conserved miRNA” and the “Good mirSVR score, Non-conserved miRNA” were downloaded from www.microrna.org/microrna/ getDownloads.do. Interactions were subsequently merged into a single list and duplicated interactions were removed. To increase confidence, we eliminated all interactions that had a mirSVR score higher than -0.1 following the recommendation given by Betel.[7] This

produced a list of 92141 miRNA-RNA interactions.

- **Pita:** Mouse miRNA targets were obtained from the Download Page (https://genie.weizmann.ac.il/pubs/mir07/catalogs/PITA_sites_mm9_0_0_ALL.tab.gz). A minimal score of -10 was set according to the authors' indications in the FAQ/Notes page (https://genie.weizmann.ac.il/pubs/mir07/index.html). Applying this threshold, we obtained 19615 miRNA-mRNA interactions.
- **miRDB:** Predicted interactions were retrieved from the miRDB webportal (http://www.mirdb.org/miRDB/download/miRDB_v5.0_prediction_result.txt.gz). Following the recommendations stated on the Download page, a gene with a score higher that 50 should be considered as a likely miRNA target. Considering only interaction with a score higher than 50 resulted in 68035 miRNA-mRNA interactions.
- **TargetScan***:* Murine miRNA-mRNA interactions were downloaded from the TargetScan webpage (http://www.targetscan.org/mmu_71/). Only interactions with "Cumulative weighted context++ score" lower than -0.1 were to increase prediction confidence as suggested by Agarwal *et al.* (2015) resulting in 18612 interactions.
- **MirTarBase:** miRNA-mRNA interactions for mouse based on experimental data were downloaded from MirTarBase Version 6.1 (http://mirtarbase.mbc.nctu.edu.tw/cache/download/6.1/mmu_MTI.xls). The complete set of 13027 miRNA-mRNA interactions of the MirTarBase was included in HeartmiR.

For integration with the expression data, miRNA targets were mapped to their gene symbol and Entrez Gene ID. We only kept interactions for which the corresponding mRNAs and miRNAs have higher expression than 32 units for at least one time point. To facilitate assessment of predicted interactions and comparison of their scores, we ranked the scores and converted them to percentiles for each resource separately. This information is displayed when using HeartmiR.

c. Use of HeartmiR

HeartmiR can be queried for interactions with given miRNA and genes or for specific miRNA-mRNA interactions. Several gene or miRNA identifiers can be used to define the query. Currently, accepted gene identifiers include the gene name, gene symbol, Entrez Gene ID (according to NCBI annotation) and the corresponding Affymetrix ID. Identifiers for miRNAs that can used for querying include the miRBase ID, the Affymetrix ID and the Transcript ID. Additionally, a threshold for correlation can be set resulting in the exclusion of interactions with lower absolute correlation between miRNA and the corresponding target mRNA. For instance, setting a threshold of 0.5, only miRNA-mRNA interactions that have a Kendall correlation higher than 0.5 or lower than -0.5 will be showed. By default, the correlation threshold is set to 0.4.

The results of the query are shown in tables. For each of the queried genes and miRNAs, a separate table displays the interactions with additional information. For experimentally derived interactions, the relevant PubMed references are given. For computationally predicted interactions, a value between 1 and 100 is shown. This represents the percentile of the ranked score. The value 1 signifies that the score of the interaction is within the top 1% of all scores for the corresponding resource; a value of 2 signifies that the score is in top 1-2% and so on. Besides the Kendall correlation for the complete time

series, separate correlation coefficient for developmental time points only (“Kendall Dev”) are shown. This can enable a better examination how strongly the expression of miRNAs and their targets are (anti-)correlated during the developmental phase (E10.5-E19.5). For gene targets, the table also indicates whether they were assigned to processes related to heart development (GO:0007507) or transcription regulation (GO:0003700) in Gene Ontology. The tables can be interactively sorted and further filtered by increasing the threshold for absolute correlation. Finally, the expression profiles of miRNAs and target genes can be visualised as interactive line plots or heatmaps.

References

1. Li, X, Martinez-Fernandez, A, Hartjes, KA, Kocher, JP, Olson, TM, Terzic, A*, et al.* (2014). Transcriptional atlas of cardiogenesis maps congenital heart disease interactome. *Physiol Genomics* **46**: 482-495.
2. Chou, CH, Chang, NW, Shrestha, S, Hsu, SD, Lin, YL, Lee, WH*, et al.* (2016). miRTarBase 2016: updates to the experimentally validated miRNA-target interactions database. *Nucleic Acids Res* **44**: D239-247.
3. Agarwal, V, Bell, GW, Nam, JW, and Bartel, DP (2015). Predicting effective microRNA target sites in mammalian mRNAs. *Elife* **4**.
4. Bartel, DP (2004). MicroRNAs: genomics, biogenesis, mechanism, and function. *Cell* **116**: 281-297.
5. Kertesz, M, Iovino, N, Unnerstall, U, Gaul, U, and Segal, E (2007). The role of site accessibility in microRNA target recognition. *Nat Genet* **39**: 1278-1284.
6. Wong, N, and Wang, X (2015). miRDB: an online resource for microRNA target prediction and functional annotations. *Nucleic Acids Res* **43**: D146-152.
7. Betel, D, Wilson, M, Gabow, A, Marks, DS, and Sander, C (2008). The microRNA.org resource: targets and expression. *Nucleic Acids Res* **36**: D149-153.
