# Supplementary material for: Parallel Genome-wide Profiling of Coding and Non-coding RNAs to Identify Novel Regulatory Elements in Embryonic and Maturated Heart
Source: Mol Ther Nucleic Acids. 2018 May 4;12:158–73. doi: 10.1016/j.omtn.2018.04.018 (PMC6023836; doi:10.1016/j.omtn.2018.04.018)
Supplement: Document S2. Article plus Supplemental Information [file mmc14.pdf]

Molecular Therapy
Nucleic Acids
Original Article

Parallel Genome-wide Profiling of Coding and Non-coding RNAs to Identify Novel Regulatory Elements in Embryonic and Maturated Heart

Davood Sabour,[1,5,6] Rui S.R. Machado,[2,6] José P. Pinto,[2] Susan Rohani,[1] Raja G.A. Sahito,[1] Jürgen Hescheler,[1] Matthias E. Futschik,[2,3,4] and Agapios Sachinidis[1]

[1]University of Cologne (UKK), Institute of Neurophysiology and Center for Molecular Medicine Cologne (CMMC), Robert-Koch-Str. 39, 50931 Cologne, Germany; [2]Systems Biology and Bioinformatics Laboratory (SysBioLab), Center for Biomedical Research (CBMR), University of Algarve, Campus de Gambelas, 8005-139 Faro, Portugal; [3]Centre of Marine Sciences (CCMAR), University of Algarve, 8005-139 Faro, Portugal; [4]School of Biomedical Sciences, Faculty of Medicine and Dentistry, Institute of Translational and Stratified Medicine (ITSMED), University of Plymouth, Plymouth PL6 8BU, UK; [5]Department of Genetics, Faculty of Medicine, Babol University of Medical Sciences, 47134 Babol, Iran

Heart development is a complex process, tightly regulated by numerous molecular mechanisms. Key components of the regulatory network underlying heart development are transcription factors (TFs) and microRNAs (miRNAs), yet limited investigation of the role of miRNAs in heart development has taken place. Here, we report the first parallel genome-wide profiling of polyadenylated RNAs and miRNAs in a developing murine heart. These data enable us to identify dynamic activation or repression of numerous biological processes and signaling pathways. More than 200 miRNAs and 25 long non-coding RNAs were differentially expressed during embryonic heart development compared to the mature heart; most of these had not been previously associated with cardiogenesis. Integrative analysis of expression data and potential regulatory interactions suggested 28 miRNAs as novel regulators of embryonic heart development, representing a considerable expansion of the current repertoire of known cardiac miRNAs. To facilitate follow-up investigations, we constructed HeartMiR (http://heartmir.sysbiolab.eu), an open access database and interactive visualization tool for the study of gene regulation by miRNAs during heart development.

INTRODUCTION

Heart development comprises a series of temporally and spatially coordinated processes, involving distinct cell populations.[1] In mice, a primitive linear heart tube is formed from the lateral mesoderm at embryonic day 8 (E8.0). As the embryo continues to develop, chamber formation and septation lead to formation of a functional four-chambered heart.[2] Key cardiac transcription factors (TFs), including Tbx5, Nkx2-5, Gata4, Isl1, and Mef2c, have been identified and their roles in heart development extensively studied (for review, see Meganathan et al.[8]).[3–8] These TFs form a conserved network and drive differentiation of cardiac progenitor cells toward cardiomyocytes, cardiac endothelial cells, and other cell types that constitute a functional heart. In addition, extrinsic signaling molecules, such as bone morphogenic proteins (BMPs) in the anterior lateral plate mesoderm, are essential for the initiation of myocardial differentiation and cardiac developmental processes.[9] More recently, small non-coding RNAs, termed microRNAs (miRNAs) have been recognized as key regulators of organ development in several organisms, such as *Caenorhabditis elegans*, *Drosophila melanogaster*, and humans.[10–13]

miRNAs (21–25 nt in length) commonly regulate gene expression at post-transcriptional level through imperfect base pairing to target mRNAs,[14] leading to translational inhibition and mRNA degradation.[15] Formation of mature miRNAs occurs in four steps: (1) transcription of a primary miRNA (pri-miRNA) via RNA polymerase II; (2) processing of the pri-miRNA by Drosha (a RNase III type endonuclease) and the double-stranded RNA binding protein DGCR8 (DiGeorge syndrome critical region gene 8) complex to produce a hairpin precursor miRNA (pre-miRNA), consisting of ∼70 nt in the nucleus; (3) export of the pre-miRNA to the cytosol via the protein exportin-5; and (4) cleavage by Dicer (a the RNase III type endonuclease) to produce ∼22-bp double-stranded miRNA. Following this final step, one strand is typically degraded, whereas the other strand is integrated into the RNA-induced silencing complex (RISC). The RISC complex targets complementary sequences in the mRNAs that are mainly located in the 3′ UTR but can also be found in the 5′ UTR and coding region, resulting in degradation of the mRNA or inhibition of its translation.[13]

Received 12 September 2017; accepted 30 April 2018;
https://doi.org/10.1016/j.omtn.2018.04.018.

[6]These authors contributed equally to this work.

Correspondence: Agapios Sachinidis, University of Cologne (UKK), Institute of Neurophysiology and Center for Molecular Medicine Cologne (CMMC), Robert-Koch-Str. 39, 50931 Cologne, Germany.
E-mail: a.sachinidis@uni-koeln.de
Correspondence: Matthias E. Futschik, School of Biomedical Sciences, Faculty of Medicine and Dentistry, Institute of Translational and Stratified Medicine (ITSMED), University of Plymouth, Plymouth PL6 8BU, UK.
E-mail: matthias.futschik@plymouth.ac.uk

This is an open access article under the CC BY license (http://creativecommons.org/licenses/by/4.0/).

A number of studies have suggested crucial roles for miRNAs in heart development and function. For instance, overexpression of miR-1 in a developing mouse heart inhibits proliferation of ventricular cardiomyocytes, causing developmental arrest at E13.5, as a result of thinning of the ventricle walls and heart failure.[14] Meanwhile, overexpression of the miR-17-92 cluster induced proliferation of both neonatal and adult cardiomyocytes. Overexpression of this miRNA cluster in adult cardiomyocytes also protected the heart from myocardial-infarction-associated injury, potentially through reducing the expression of phosphatase and tensin homolog (Pten)—a proliferation repressor.[16] Moreover, miRNA dysregulation has been associated with various cardiac events, influencing myocardial structure, contractility, fibrosis, and apoptosis.[17] Despite evidence of the impact of miRNAs on heart development and functional integrity, no comprehensive profiling of their expression during cardiogenesis has been undertaken to date. To address this salient lack of knowledge, we performed parallel genome-wide profiling of miRNAs and polyadenylated [poly(A)]-RNAs from developing hearts of mice embryos from E10.5 until E19.5, as well as from mature hearts of young and old adult mice. Profiles of poly(A)-RNA and miRNA were analyzed independently, and their differential expression was determined. Gene clustering analysis revealed a distinct temporal activation during the developmental processes, indicating specific genes involved in cardiogenesis. Integrative analysis of gene expression profiles and gene targets led to prioritization of 27 miRNAs as novel potential regulatory factors of heart development. Finally, an open access database and interactive visualization tool, called HeartMiR (http://heartmir.sysbiolab.eu), was developed to identify (anti-) correlated expression patterns of miRNAs and their target genes during heart development and in mature heart tissue. This database provides a resource for cardiac gene regulation by miRNAs and a basis for further investigation of heart development, congenital heart diseases, or cardiac regenerative medicine.

RESULTS

Parallel Profiling of the poly(A)-RNA and miRNA Transcriptomes

To assess dynamic expression of poly(A)-RNA and miRNA during murine heart development *in vivo*, tissue samples from embryonic hearts were taken each day from E10.5 to E19.5 (Figure 1A). For comparison, we also collected samples from young and old adult hearts. Microarray experiments were performed for triplicate biological samples of both poly(A)-RNA and miRNA. Notably, heart tissue was dissected from both male and female embryos and adults to identify any gender-specific effects on gene expression. Seventy-two samples were profiled using Affymetrix Mouse Genome 430 2.0 Arrays and Affymetrix miRNA 3.0 GeneChips. Although the majority of probes on the Affymetrix Mouse Genome platform target mRNAs, this platform also included probes against numerous long non-coding RNAs (lncRNAs).

For both poly(A)-RNAs and miRNAs, clustering of the full microarray profiles was carried out. In general, samples from the same developmental status were grouped, suggesting a robust temporal expression signature (Figure S1). Expression profiles of young and old adult hearts formed a distinct cluster. Similar patterns also emerged through principal-component analysis (PCA). Global expression during development (E10.5–E19.5) gradually approximated the mature heart, although a clear segregation of embryonic and mature samples remained (Figures 1B and 1C). This indicates substantial differences in expression between developing and mature cardiac tissue. Interestingly, the distribution of poly(A)-RNA levels for E10.5–E13.5 showed a notable "shoulder," suggesting an underlying bimodal distribution, whereas samples from later time points displayed a gradual decrease in the number of genes with high expression values (Figure S2). Such bimodality might suggest a greater tendency toward an "on or off" mode of expression during early development, with more gradual adjustment during later stages of development.

Genes Linked to Heart Development and Function Show Distinct Expression Changes

To detect differentially expressed genes (DEGs) transcripted as poly(A)-RNAs, normalized Affymetrix GeneChip signal intensities of various developmental stages were compared with intensities for young adult hearts. Because a large number of genes displayed changes in expression, a very stringent threshold for differential expression was set, using an adjusted p value of $\leq 10^{-5}$ and an absolute log2 fold change of ≥ 2 (4-fold change). Collectively, we found 2,708 non-redundant genes to be differentially expressed for at least one time point. These represent 13% of the genes covered by the array. Notably, the number of DEGs reduced drastically with ongoing development, reducing to 2,080 at E10.5 and further to 495 at E19.5 (Figure 1D). DEGs at each time point are listed in Table S1. To evaluate the dynamics of gene expression during cardiogenesis, we initially inspected the transcript levels of genes associated with heart development in Gene Ontology (GO: 0007507; Table S2). TFs essential for heart development, such as *Gata3*, *Gata4* (Figure 2A), *Nkx2-5*, and *Hand2*, were gradually downregulated during embryonic development, whereas *Myocd* (transcriptional co-activator of serum response factor) displayed an initial increase in expression but plateaued from E15.5 to E18.5 (Figure 2B). All these TFs were only weakly expressed in mature hearts. Expression of *Foxc1*, *Foxc2*, and *Foxp1* of the forkhead family of TFs, known to play an important role in embryonic heart development,[18] decreased with progressive development, reaching lowest levels in adult tissue (Figure 2C). Consistent with the established role of BMPs in transforming growth factor beta and Wnt signaling pathways during cardiac development,[9] the expression of *Bmp2*, *Bmpr1a* (bone morphogenetic protein receptor type 1A), *Tgf-β*, and *Wnt5a* was downregulated over time (Figure 2D). Next, we inspected expression of genes associated with contraction of cardiac muscle, affecting the primary function of the heart (Figure 2E). Strikingly, cardiac alpha actin gene (*Actc1*), a known marker for early myogenesis,[19] had the highest signal intensity of all genes at E10.5 but was subsequently downregulated, having minimum expression in the adult heart. Likewise, *Myh7*, which encodes for the β-myosin heavy chain, was highly expressed at all developmental stages but weakly expressed in adult hearts. This finding is consistent with previous observations of postnatal downregulation of *Myh7* in mice and other

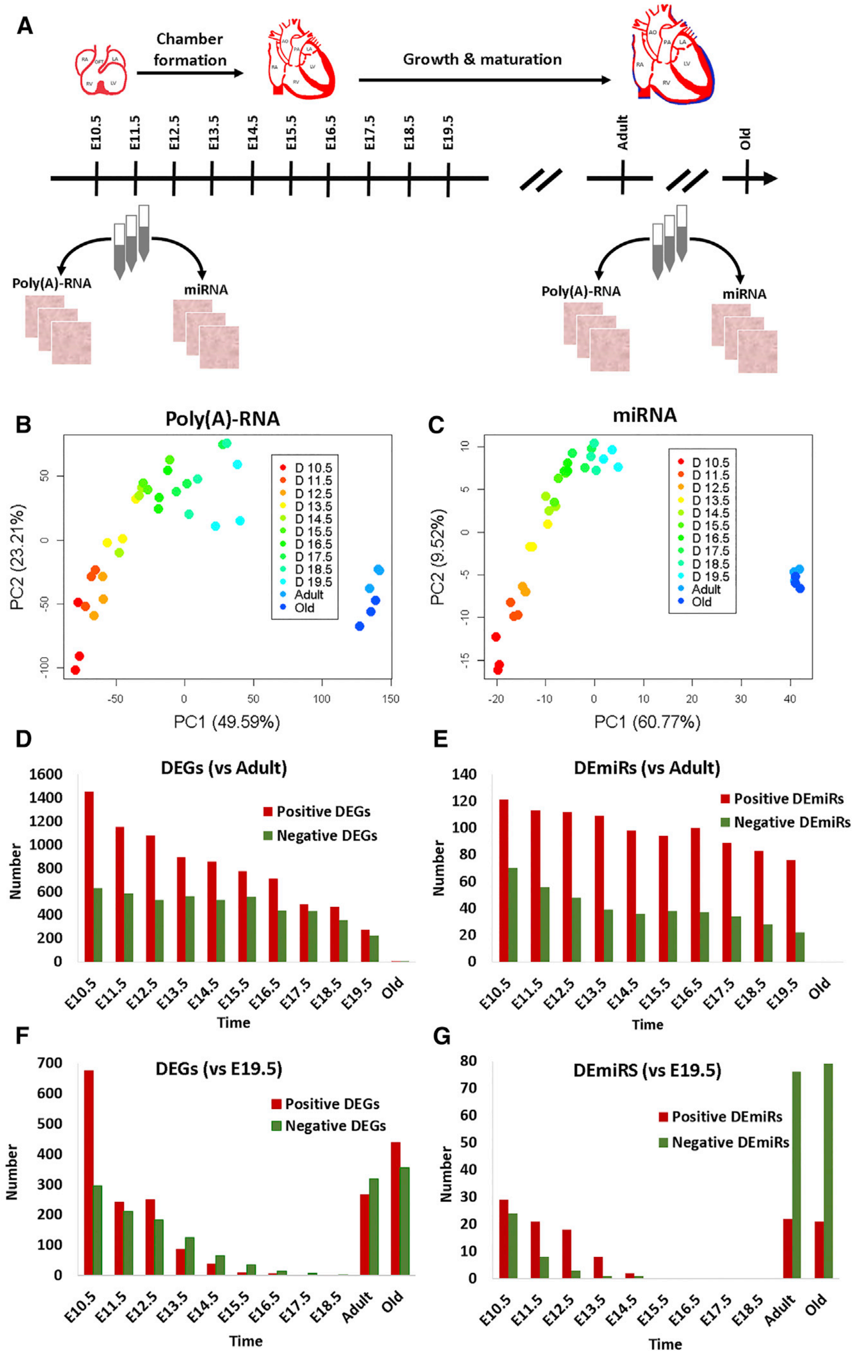

Figure 1. Experimental Design, Principal-Component Analysis of Transcriptome Data, and Number of Differentially Expressed Genes and miRNAs

(A) Tissue samples from the developing heart at time points from E10.5 to E19.5, as well as from young and old adult hearts, were taken, and the extracted RNA was analyzed by microarray experiments. (B) Principal-component analysis of the expression of genes coding for poly(A)-RNA is shown. (C) Principal-component analysis of miRNAs is shown. (D and E) Number of differentially expressed genes (D) and miRNAs (E) in developing heart and older adult heart tissues, compared with young mature heart tissue, is shown. (F and G) Number of differentially expressed genes (F) and miRNAs (G) in developing heart and older adult heart tissues, compared with E19.5 heart tissue, is shown.

rodent hearts.[20] In contrast, the expression of cardiac troponin I (*Tnni3*) was gradually upregulated during development, with maximum expression occurring in mature hearts. An even more extreme case, showing almost switch-like upregulation, was observed for titin-cap (*Tcap*), linked to sarcomere assembly. Throughout most of the monitored developmental stages, it was expressed at marginal levels but began to accumulate at E19.5 only and was more than 10-fold upregulated in adult hearts. We also investigated expression in epigenetic regulators. In particular, expression of histone deacetylase 2 (*Hdac2*) strongly reduced in a linear manner during development, exhibiting a greater than 10-fold change (Figure 2F). *Hdac2* deacetylates lysine residues at the N-terminal regions of the core histones H2A, H2B, H3, and H4, playing an important role in transcriptional regulation and plasticity.[21] Table S3 indicates differentially expressed genes associated with the activity of ion channels in GO identified in our study. Close scrutiny suggests that ion channels undergo major remodeling during cardiac development. For instance, *Cacna1h* and *Cacna2d2* encoding for the α_1- and α_2-δ subunits of voltage-gated calcium channels, respectively, were gradually downregulated during development and were expressed at relatively low levels in adult hearts. In contrast, the gene *Cacna1g*, which is a paralog of *Cacna1h* that encodes for an alternative α_1 subunit, was initially upregulated, reaching a maximal plateau during late developmental stages (Figure 2G). Among the genes corresponding to sodium channels, we found that the α subunit encoded by the *Scn7a* gene remained very weakly transcribed in embryonic hearts but had up to 10-fold higher expression in mature hearts, displaying a switch-like expression pattern (Figure 2H). The genes for potassium channels, *Kcna5* and *Kcnb1*, were very lowly expressed in all developmental stages but induced in both young and old adult hearts (Figure 2I). In contrast, transcripts of *Kcne1* gradually accumulated during development but were markedly depleted in adult hearts (Figure 2J). Expression of *Kcnq5* tended to be more strongly repressed as development occurred, whereas expression of *Kcna4* showed a parabolic expression pattern, with maximal expression at E15.5

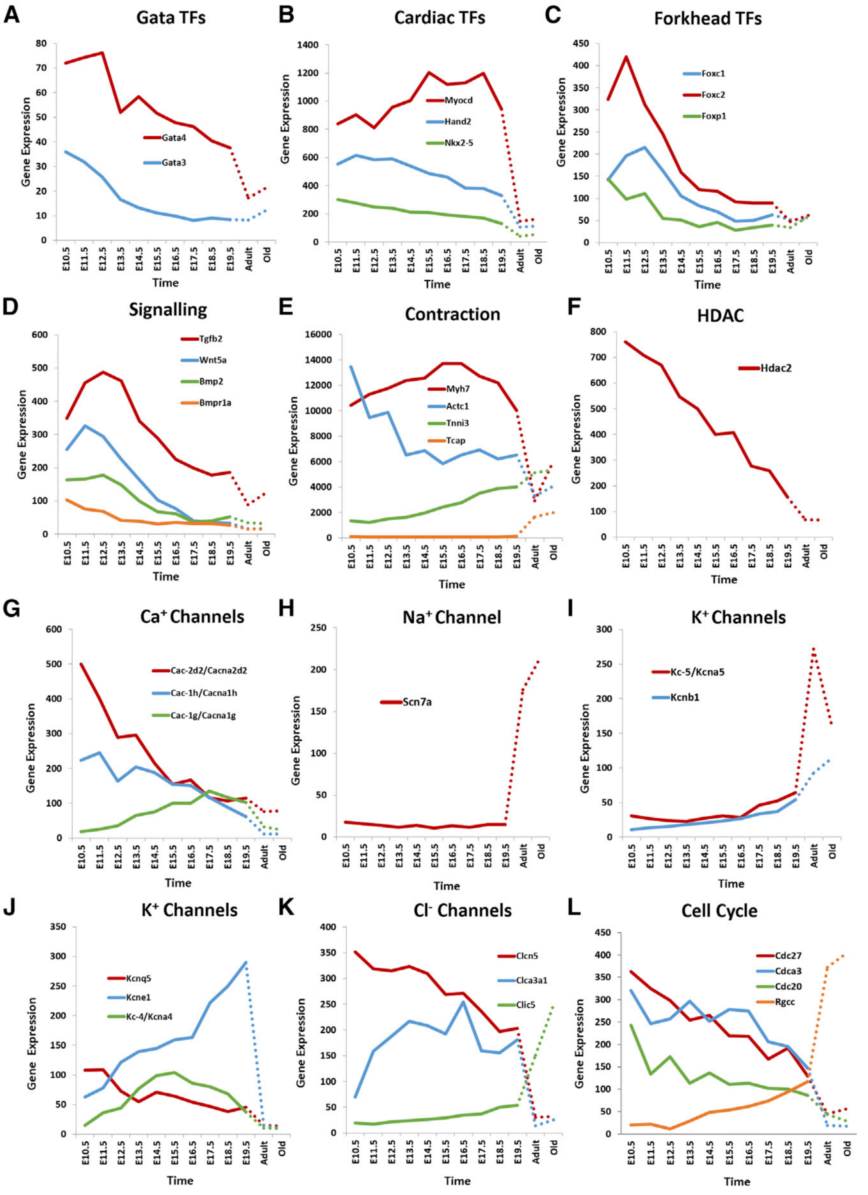

Figure 2. Temporal Expression Profiles of Selected Genes with Existing Association with Heart Development and Ion Channel Function

(A) Gata transcription factors (TFs). (B) Known key TFs for cardiogenesis are shown. (C) Fox family TFs are shown. (D) Signaling pathway molecules linked for cardiac development are shown. (E) Genes encoding for structural cardiac proteins are shown. (F) Histone deacetylase is shown. (G) Calcium channel genes are shown. (H) Sodium channel gene is shown. (I and J) Potassium channel genes upregulated (I) and downregulated (J) in young mature heart tissue are shown. (K) Chloride channel genes are shown. (L) Cell cycle genes are shown. Dotted lines were plotted between E19.5, young, and old adult stages to emphasize the largely increased time intervals between these time points compared to the previous time points.

(Figure 2J). A remarkable transcriptional plasticity was also observed for chloride (Cl^{-}) channels. Expression of *Clinc5* progressively decreased between E10.5 and E19.5, resulting in very low levels in adult hearts (Figure 2K). A transient pattern was observed for *Clca3a1*. After an initial increase from E10.5 to E13.5, an almost stable expression level was maintained until E19.5 before a drastic reduction occurred, yielding very low levels in both young and old adult hearts. In contrast, *Clic5*, which encodes for a chloride channel located in cardiomyocyte mitochondria of different rodents,[22] displayed a linear increase in expression during development, with a considerable boost in expression in adult heart tissue. Finally, the transcript level of cell division cycle (Cdc) genes, such as *Cdc27*, *Cdc20*, and *Cdca3*, decreased during embryonic development and were further reduced in mature cardiac tissue, reflecting the ceasing of cell cycle activity with progressing heart development. Interestingly, we found that *Rgcc* (regulator of cell cycle) displayed a contrasting pattern with low transcript levels during earlier development but high levels in adult heart. This suggests that *Rgcc* might contribute to postnatal cell cycle arrest in cardiomyocytes.

To assess the reliability of the generated transcriptomics data, we compared them with those reported in a related microarray study of murine heart development.[23] A cross-correlation analysis verified both time series studies were in concordance on the transcriptome level (Supplemental Information; Figure S3).

Gene Expression of Transcripts of Unknown Function during the Heart Development and Adult Heart

We were especially interested in genes listed as Riken genes or predicted in the Mouse Genome Informatic (MGI) database. The former originate from sequencing of full-length cDNA libraries collected at the Riken Institute, and many of these remain poorly characterized. In our study, we found 127 Riken and predicted genes among the DEGs. After removal of genes functionally annotated in GO, 107 DEGs remained (Table S4). Remarkably, almost a quarter (23%) was curated as (long) non-coding RNA genes in the MGI database.

We assigned protein-coding Riken and predicted genes to three classes, based on whether their expression patterns showed (1) a gradual decrease in expression over time (29 genes; Figure 3A); (2) a transient higher expression during development (37 genes; Figure 3B); or (3) a gradual increase in expression over time (16 genes; Figure 3C). A particularly strong increase of over 6-fold change and

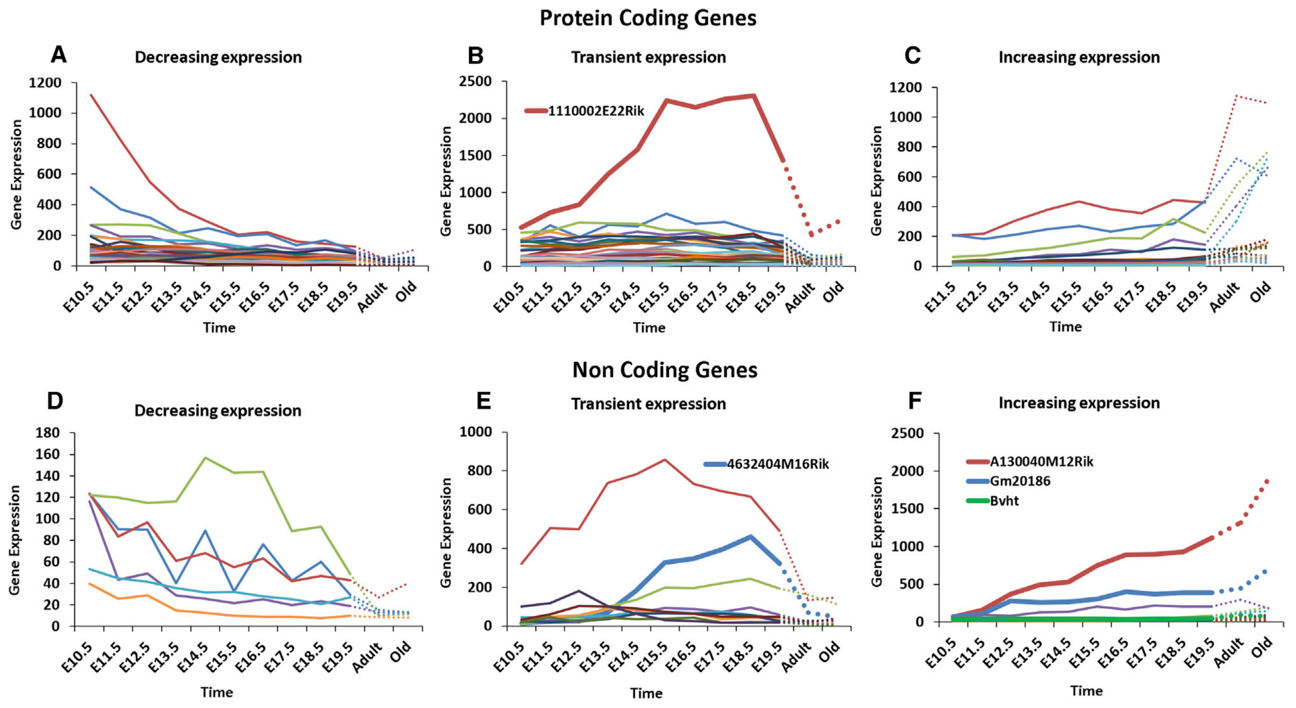

Figure 3. Differentially Expressed Genes Encoding for Poly(A)-RNA without Current Functional Annotation

(A–C) Protein-coding genes with decreased (A), transient (B), or increased (C) expression. (D–F) Non-coding genes with decreased (D), transient (E), or increased (F) expression during embryonic development are shown.

a high absolute expression level at E15.5 was recorded for *1110002E22Rik*. This was also one of the few Riken genes, for which a putative protein domain was detected. Close to the C terminus, a histone deacetylase superfamily domain (IPR000286) was predicted by Interpro, providing an interesting clue to the function of *1110002E22Rik*.

Similarly, the differentially expressed non-coding genes were grouped into three classes, reflecting their expression patterns. In this case, six genes were downregulated during development (Figure 3D) and ten genes displayed transiently higher expression (Figure 3E), whereas nine genes were gradually induced (Figure 3F). Two lncRNAs showed an especially strong increase in expression during development: Riken *A130040M12* (29-fold) and *Gm20186* (10-fold). The former has a 3,366-nt transcript derived from Viral-like 30 elements (VL30s). The later leads to a 741-nt transcript and was detected as widely expressed across multiple organs in mouse embryos by *in situ* hybridization (MGI-Gene Expression database). The expression of lncRNAs increased in old compared with young adult hearts. A similar expression pattern was found for Braveheart, an lncRNA that was recently shown to modulate the expression of cardiac TFs. Whereas Braveheart was not included in the list of DEGs because of our stringent threshold, its previously reported expression is consistent with our data.[24] In our study, Braveheart displayed low transcript levels during developmental stages but was two-fold upregulated in mature heart tissue (Figure 3F). A more transient expression pattern was observed for *4632404M16Rik* (Figure 3E), which is an intronic lncRNA of 2,882 nt length. It initially follows the upregulation of cardiac calsequestrin 2 (*Casq2*), in which *4632404M16Rik* is located. CASQ2 is the most abundant Ca^{2+}-binding protein in the sarcoplasmic reticulum and integral to the high Ca^{2+} capacity of the sarcoplasmic reticulum. Although *Casq2* remained highly expressed after development, *4632404M16Rik* expression was only weak in mature tissue, suggesting that its function is evoked mainly during cardiogenesis (Figure S4).

Genes Expressed in an Age- or Gender-Dependent Manner

PCA and clustering analysis (Figures 1B and S1) indicate that gene expression in the hearts of older adult mice (10 months old) closely resembles that in hearts of younger adult (10 weeks old) mice. Only seven genes were found to be differentially expressed (Table S1). The most upregulated gene in older hearts was *Adamts9*, which is a member of the ADAMTS (a disintegrin and metalloproteinase with thrombospondin motifs) family. It encodes for a secreted protease, acting as an angiogenesis inhibitor.[25] The most downregulated gene was the coiled-coil domain containing 141 (*Ccdc141*).

Over our time series, only three genes were consistently differentially expressed between male and female tissue samples. As expected, *Xist* was detected only in female samples, whereas *Eif2s3y* and *Ddx3y*, both located on the Y chromosome, were detected only in male samples.

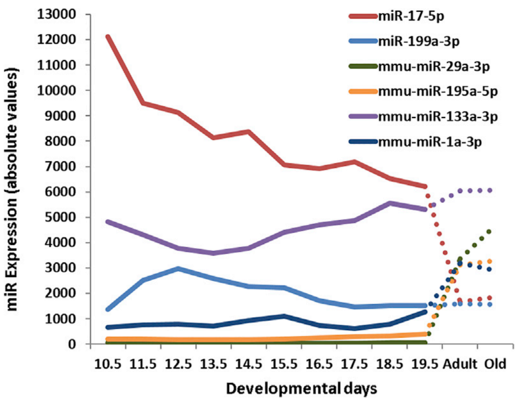

Figure 4. Temporal Expression Profiles of Selected DEmiRs for Embryonic and Mature Heart Tissues

Clearly, the extent of gender-specific expression was marginal during development, although we observed significantly larger variability in expression between male and female samples in adult samples, especially in older mature heart tissue (Figure S5). The number of genes having more than a 2-fold expression change between male and female samples increased from five at E19.5 to thirty in adult tissue and up to 98 in older mature tissue (Table S5). Of these, 55 genes were upregulated and 43 were downregulated in male heart tissue. A GO enrichment analysis of these upregulated genes showed significant overrepresentation of cytoskeletal genes, especially those associated with actin filament and organization. The most upregulated gene was gelsolin, which encodes for a protein regulating the actin cytoskeleton. In contrast, no enriched GO categories were associated with downregulated genes.

Differential miRNA Expression during Heart Development

The same method and thresholds for DEGs were used to identify differentially expressed miRNAs (DEmiRs). We identified 217 DEmiRs in this study. Similar to DEGs, the number of DEmiRs gradually decreased from 191 at E10.5 to 98 at E19.5, as heart development progressed (Table S6; Figure 1E). Comparing the number of upregulated and downregulated DEmiRs, we found 2.5–3.5 times more upregulated than downregulated DEmiRs during heart development. This contrast with the ratio of upregulated to downregulated DEGs, which is more balanced in later stages of development (Figure 1D). Among the DEmiRs, we identified a number of miRNAs associated with the cardiac cell lineage, including miR-17, miR-29-3p,[26,27] miR-199a-3p,[28,29] miR190-3p,[30] and miR-1.[31–33] The expression of miR-17-5p was gradually downregulated from E10.5 to E19.5, with a subsequent 3-fold drop in expression in adult heart tissue (Figure 4). In contrast, the expression levels of miR-29a-3p, miR-195a-5p, and miR-1a were relatively low during all developmental stages but highly upregulated in adult hearts. More transient expression patterns were displayed by miR-199a-3p and miR-133a, for which we recorded maximum and minimum signal intensities at developmental stages of E12.5 or E13.5, respectively. As in the case of poly(A)-RNAs, we searched for differentially expressed miRNAs in older versus younger adult heart tissue, as well as for gender-specific changes. However, no significant differential expression was detected in these comparisons.

Dynamics of Gene Expression during Embryonic Heart Development

Our approach to use the young heart tissue as reference delivered a large number of genes that were differentially expressed in embryonic compared to mature tissue. To demarcate genes that showed significant expression changes during the embryonic development, we reanalyzed the transcriptomic with E19.5 as new reference. As shown in Figures 1F and 1G, the number of the newly detected DEGs and DEmiRs rapidly decreased in the early stages of the development (E10.5–14.5) in exponential manner. Compared to the higher large numbers of genes and miRNAs observed in the previous comparison (Figures 1D and 1E), the numbers of genes and miRNAs with significant dynamic expression during development were considerably smaller. In particular, only a few genes and no miRNAs were found differentially expressed for E15.5–E18.5. Analyzing the expression in mature tissue with E19.5 as reference confirmed the striking imbalance between positively and negatively regulated miRNAs. We identified 3.5 times more negatively regulated than positively regulated miRNAs in mature tissue when compared to E19.5. Tables S7 and S8 list the genes that were found differentially regulated at the different heart developmental stages using the E19.5 heart developmental stage as a reference in comparison to young adult heart applied as a reference, respectively. Tables also show commonly differential expressed genes and miRs identified by applying the adult and E19.5 developmental stage as references. Moreover, we have also identified non-uniquely and uniquely genes (Table S9) and miRs (Table S10) that were expressed for each developmental stage.

Integrative Analysis of Dual Transcriptome Data and miRNA Gene Targets

To obtain comprehensive coverage of potential miRNA targets, we integrated miRNA interactions from five independent resources (see Materials and Methods). Stringent filtering procedures were applied to predicted miRNA interactions to reduce the number of false positives (see Supplemental Information). We only included interactions in which the miRNA or its target was expressed at least at one time point in this study. This led to compilation of 102,083 potential interactions between 368 miRNA and 9,211 gene targets, covered by our microarray platforms. To cope with this complexity and evaluate major expression trends, we clustered the DEGs and DEmiRs separately, based on their standardized expression profiles using fuzzy c-means clustering.[34] This resulted in the detection of six clusters of DEGs and three clusters of DEmiRs. Each possible pair of clusters of DEGs and DEmiRs was subsequently evaluated for putative miRNA-gene interactions to derive a regulatory interaction matrix (M_{all}). Because miRNAs are commonly considered to be post-transcriptional repressors, we also determined an interaction matrix M_{neg}, which included only anti-correlated interactions between miRNAs and their

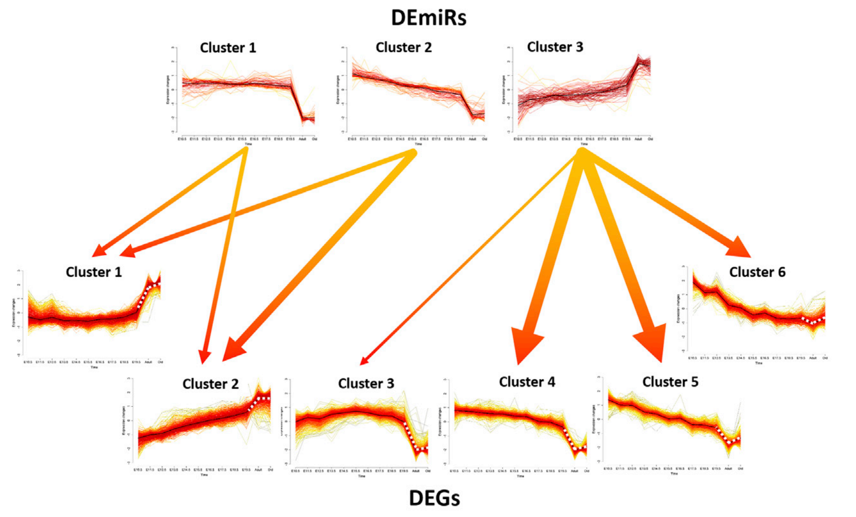

M_{all}	mRNA Cl1	mRNA Cl2	mRNA Cl3	mRNA Cl4	mRNA Cl5	mRNA Cl6
miRNA Cl1	241	179	188	377	342	281
miRNA Cl2	248	198	207	393	396	280
miRNA Cl3	231	168	188	365	338	267

M_{neg}	mRNA Cl1	mRNA Cl2	mRNA Cl3	mRNA Cl4	mRNA Cl5	mRNA Cl6
miRNA Cl1	109	103	4	4	8	4
miRNA Cl2	117	180	6	1	2	2
miRNA Cl3	4	0	70	264	275	181

Figure 5. Integrative Analysis of Clusters of DEmiRs and DEGs

The fuzzy c-means algorithm was used to obtain three DEmiR clusters and six DEG clusters. Clusters were linked based on miRNA-target gene interactions. Links are only displayed when dominated by negatively correlated miRNAs and target gene interactions. The tables in the bottom panel display the total number of potential regulatory interactions between pairs of DEmiR and DEG clusters (M_{all}) and the number of interactions between negatively correlated miRNAs and target genes (M_{neg}). The width of the arrow indicates the number of anti-correlated targeted genes.

target genes. In this way, we combined our clustering approach and interaction data to generate a compact network of miRNA and gene clusters displayed in Figure 5, together with the regulatory interaction matrices M_{all} and M_{neg}. Given that, in classical models, miRNA binding leads to repression of their targets, we focused on the links between DEmiR and DEG clusters, in which putative negative regulatory interactions dominated.

DEmiRs in cluster 1 were strongly downregulated in mature heart tissue compared with samples of developing hearts. Only minor differences in their expression levels were observed between stages of embryonic development. Compared with DEmiR cluster 1, DEG cluster 1 showed the strongest anti-correlation in expression. Accordingly, DEG cluster 1 showed little change in gene expression from E10.5 to E19.5 but a considerable increase in mature tissue. Remarkably, miRNAs of DEmiR cluster 1 targeted 241 of the 643 genes included in DEG cluster 1. We also detected significant enrichment of target genes among biological processes associated with metabolism, such as oxidation-reduction processes (false discovery rate [FDR] = 2.0E−5) and the immune system (FDR = 0.009).

DEmiR cluster 2 showed a gradual decrease in expression during embryonic development and further downregulation in mature heart tissue. These changes in expression levels suggest a dynamic adjustment of the regulatory function of miRNAs included in DEmiR cluster 2 during embryonic development and are in contrast to miRNAs included in DEmiR cluster 1, which showed little expression change over this period. DEG cluster 2 displayed anti-correlated gene expression behavior with DEmiR cluster 2, having a gradual increase in gene expression during embryonic heart development and further upregulation in mature tissue. Notably, a large number of genes in DEG cluster 2 were targeted by miRNAs in DEmiR cluster 2, i.e., 198 out of 517 genes. Further functional enrichment analysis of the targeted genes in DEG cluster 2 revealed a significant association with developmental biological processes, including angiogenesis (FDR = 3.7E−5) and cardiovascular system development (FDR = 9.9E−6).

Finally, the expression of most miRNAs in DEmiR cluster 3 increased only during embryonic heart development but reached high expression levels in mature hearts. DEG clusters 3, 4, 5, and 6 showed the opposite pattern of gene expression over time, comprising genes with decreasing expression during heart development and minimum expression in young and old adult hearts. Members of DEmiR cluster 3 targeted 188 genes belonging to DEG cluster 3, 365 genes of DEG cluster 4, 338 genes of DEG cluster 5, and 267 genes of DEG cluster 6. GO analysis of the targeted DEGs from clusters 3, 4, 5, and 6 indicated statistically enriched biological processes, such as regulation of gene expression, cell cycle (FDR = 7.3E−5), heart development (FDR = 2.8E−5), and cytoskeleton organization (FDR = 0.0006). These results suggest that members of cluster 3 DEmiRs act as key regulators of cardiogenesis, given their potential to induce progressive downregulation of genes that were especially active during development and postnatal maturation.

Alternative to the data-driven clustering approach connecting the temporal profiles of DEGs and DEmiRs, we also aimed to link individual miRNAs to specific genes that are known to be involved in heart development and that we examined earlier (Figure 2). To this end, we searched for miRNAs that target these genes and show anti-correlated expression during embryonic development. We excluded mature samples in the calculation of correlation to avoid confounding effects through large postnatal expression changes that frequently were observed. Table 1 displays anti-correlated miRNAs that target genes in the specific sub-categories shown in Figure 2.

Table 1. Highly Anti-correlated miRs to the Specific Gene Subcategories in Figure 2

		Kendall Dev
Gata TFs		
Gata4	miR-3474	−0.4
	miR-22-5p	−0.26
Gata3	miR-27a-3p	−0.64
	miR-34a-5p	−0.4
Cardiac TFs		
MyoCD	miR-495-3p	−0.6
	miR-31-3p	−0.54
Hand2	miR-671-5p	−0.26
	miR-181a-5p	−0.18
Nkx2-5	miR-207	−0.02
Forkhead TFs		
Foxc1	miR-133b-3p	−0.56
	miR-680	−0.46
Foxc2	miR-133a-3p	−0.61
	miR-133b-3p	−0.61
Foxc3	–	–
Signalling		
Tgfb2	miR-301a-3p	−0.57
	miR-193b-3p	−0.52
Wnt5a	miR-378a-5p	−0.71
	miR-378b	−0.66
Bmp2	let-7b-5p	−0.48
	miR-27a-3p	−0.44
Bmpr1a	miR-194-5p	−0.72
	miR-362-3p	−0.46
Contraction		
Myh7	–	–
Actc1	miR-30a-5p	−0.46
	miR-30e-5p	−0.44
Tnni3	–	–
Tcap	miR-128-3p	−0.23
	miR-324-3p	−0.17
Hdac		
Hdac2	–	–
Ca^{2+} Channels		
Cacna2d2	miR-490-3p	−0.67
	miR-671-5p	−0.13
Cacna1h	miR-28a-3p	−0.63
	miR-466a-3p	−0.6
Cacna1g	miR-139-5p	−0.78
	miR-187-3p	−0.69
Na^{+} Channel		
Scn7a	–	–
K^{+} Channel		
Kcna5	miR-450b-3p	−0.2
Kcnb1	miR-409-3p	−0.8
	miR-17-5p	−0.6
Kcnq5	miR-292-5p	−0.54
	miR-324-3p	−0.41
Kcne1	miR-466g	−0.55
	miR-466f-3p	−0.42
Kcna4	miR-193b-3p	−0.46
	miR-181a-5p	−0.12
Cl^{-} Channel		
Clcn5	miR-27a-3p	−0.62
	miR-194-5p	−0.58
Clca3a1	–	–
Clic5	miR-182-5p	−0.53
	miR-485-5p	−0.45
Cell Cycle		
Cdc27	miR-362-5p	−0.46
	miR-195a-5p	−0.45
Cdca3	miR-195a-5p	−0.48
Cdc20	–	–
Rgcc	–	–

For the majority of inspected genes, potential regulatory miRNAs with a moderately or strongly anti-correlated expression could be identified. However, we did not find indications that the same miRNA targets different genes of the same functional sub-categories. Further inspection of the role of the identified miRNAs in regulating heart developmental genes is vindicated.

Temporal Expression of DEmiRs in E10.5–E19.5 Mouse Embryos

It should be noted that the inclusion of mature samples had an impact on the clustering (Figure 5). This appeared to be especially relevant for the clustering of DEmiRs. All three clusters displayed strong changes in expression between E19.5 and young mature stage, which could dominate more subtle changes during the embryonic period. Therefore, we carried out the clustering of DEmiRs without the expression for the mature tissues. This led to the detection of three new clusters (Figure 6, lower panels), which showed indeed more distinguished expression patterns during embryonic development as compared to the analysis including the mature tissues (Figure 6, upper panels; the same clusters as indicated in Figure 5A). The first cluster consisted of gradually downregulated miRs, whereas upregulated miRs were assigned to the second and the third clusters that showed distinct patterns. In the second cluster, we found miRs that were gradually

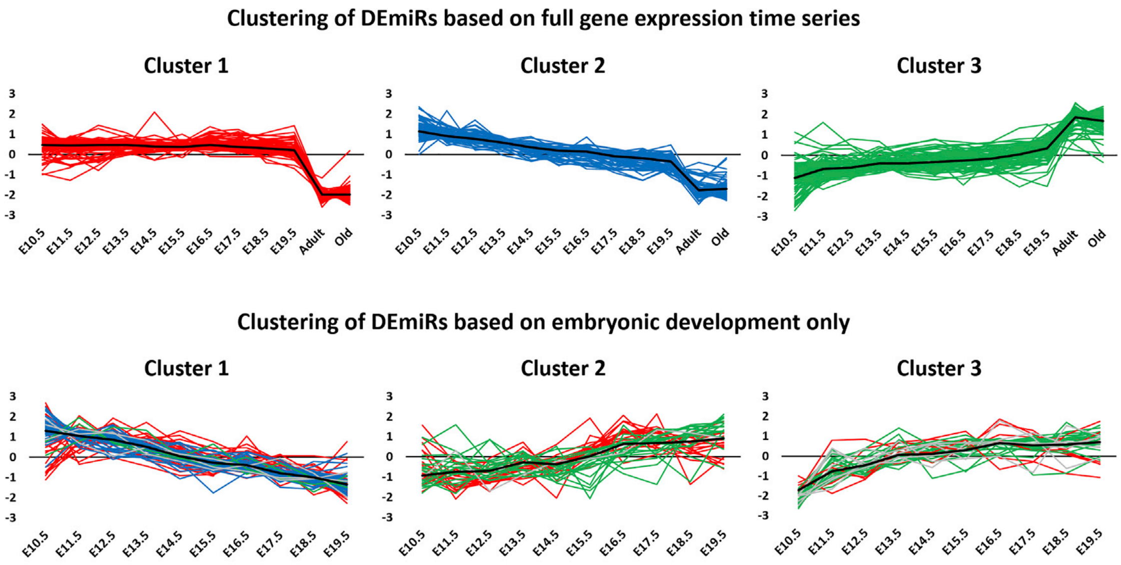

		Clustering based on full gene expression time series (including young and old adult heart)		
		Cl1	Cl2	Cl3
Clustering based on expression from E10.5 to E19.5 only	Cl1 (3 specific DEmiRs)	30	46	3
	Cl2 (1 specific DEmiRs)	17	0	18
	Cl3 (6 specific DEmiRs)	5	0	22

Figure 6. Clusters of Temporal Expression Profiles of DEmiRs for Embryonic and Mature Heart Tissues (Upper Panel) and for Only Embryonic Tissues (E10.5–E19.5) (Middle Panel)

For cluster analysis, the fuzzy c-means algorithm was used to obtain three DEmiR clusters for each of the cases. The table below shows the numbers of common DEmiRs between the two clusterings. To indicate the redistribution of DEmiRs, the coloring of the first clustering (with red for cluster 1 DEmiRs, blue for cluster 2 DEmiRs, and green for cluster 3 DEmiRs) was kept in the second clustering. DEmiRs are labeled by the light gray color in the second clustering if they were not included in the first clustering but only found with E19.5 as reference time point. The number of these DEmiRs is also included in the table. The DEmiRs belonging to the six clusters are also included in Table S6 (worksheet-cluster distribution).

upregulated, whereas the third cluster included miRs that showed the largest increase in expression at early time points and stable expression at later time points of the embryonic development.

Prioritization of miRNA Candidates Involved in Heart Development and Maturation

Our microarray experiment revealed a surprisingly large number of DEmiRs, with the vast majority of them not yet linked to heart development or cardiac tissue maturation. To assist prioritization for future studies of their functional relevance, we used several complementary approaches: (1) analysis of the correlation between miRNAs and their targets; (2) evaluation of the existing functional annotation of the targets; and (3) template-based detection of relevant miRNAs. More specifically, we classified target genes of miRNAs as negatively correlated if the Kendall's correlation coefficient of the miRNA and target gene was smaller than -0.4. Target genes were also classified as associated with heart development or with transcriptional regulation based on their current GO annotation. These data can be found in Table S11.

Assuming that the dominant mode of miRNAs is post-transcriptional repression, we would expect that target genes show a negative correlation with miRNA expression profiles. Conversely, we can evaluate the regulatory activity of a miRNA by calculating how many of its differentially expressed target genes (DETGs) are negatively correlated (DETGNCs). Plotting DETGs and DETGNCs (Figure 7A) clearly showed that many of the miRNAs having a high percentage of negatively correlated target genes are already known to be involved in the regulation of heart development or function. For instance, at least 70% of the DETGs of miR-1a-3p and miR-195a-5p (a member of the miR-15 family) were negatively correlated. This supports the conjecture that the percentage of DETGNCs could be indicative of a miRNA's regulatory activity. The highest percentage of DETGNCs ($\sim$83%) was recorded for let-7f-5p, let-7g-5p, and miR-499-5p, which belong to the so-called intronic myomiRs. Based on their high percentage of DETGNCs, we also identified miRNAs that are not yet linked to heart development, namely let-7i (82%), mir-3472 (79%), and miR-490-3p (73%; also highlighted in

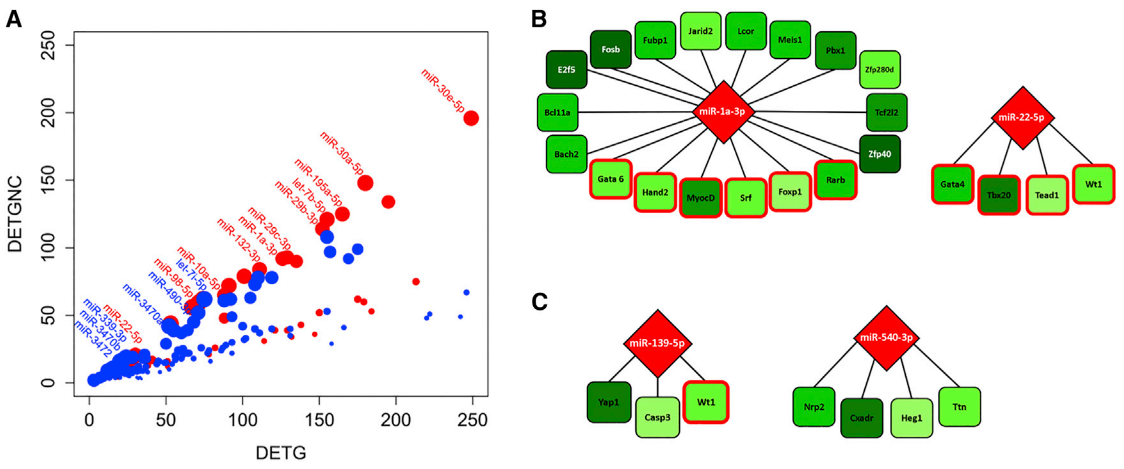

Figure 7. Prioritization of Cardiac miRNAs

(A) Number of differentially expressed targets (DETGs) and differentially expressed genes that are negatively correlated (DETGNCs). Red dots label miRNAs that have been previously associated with heart development. Blue dots label miRNAs that have not been associated with the heart development to date. Dot size indicates the percentage of anti-correlated targets, compared to all differentially expressed targets. (B) Examples of two miRNAs (symbolized as diamonds) targeting TFs are shown (squares). Red borders highlight TFs associated with heart development. The colors of the symbols indicate expression changes in young mature tissue (shades of red: upregulated; shades of green: downregulated). (C) Examples of candidates of differentially expressed miRNAs (DEmiRs) targeting differentially expressed genes (DEGs) associated with heart development are shown.

Figure 7A). These miRNAs can provide attractive leads for further studies.

In addition, we inspected the number of transcriptional regulators among the targets of miRNAs. We found 13 miRNAs that target 10 or more transcriptional regulators, with anti-correlated expression patterns. Differential expression of these miRNAs may have an especially broad effect on gene expression at a systems level. Strikingly, miR-1a-3p was the miRNA with the largest number of anti-correlated transcription regulators among its potential targets. Among the 18 transcription regulators possibly targeted by miR-1a-3p were six TFs (*Foxp1*, *Gata6*, *Hand2*, *Myocd*, *Rarb*, and *Srf*) associated with heart development, supporting a key role for miR-1 in the cardiac cell lineage (Figure 7B). Other miRNAs targeting multiple transcriptional regulators linked to heart development included miR-362-3p, miR-132-3p, miR-22-3p, miR-22-5p, and miR-221-3p. Four of the six transcriptional regulators potentially targeted by miR-22-5p are associated with heart development in GO, indicating that downstream regulatory effects of miR-22-5p may be specific to cardiogenesis (Figure 7B).

Finally, we filtered putative interactions by requiring that both miRNAs and target genes show at least a 4-fold differential expression change at one time point and were anti-correlated (Kendall correlation < -0.4). We only included target genes that were already associated with heart development in GO. Table S11 lists 51 miRNAs that were identified as targeting 48 genes annotated as heart developmental genes. Given these stringent thresholds, we retrieved only a few miRNAs that targeted several genes (e.g., miR-30e-5p targeted eight genes); the majority targeted only a single gene. However, miR-22-5p targeted both *Tbx20* and *Gata4*, i.e., two TFs that are essential for heart development (Figure 7C).[35] A review of the literature for these 51 miRNAs showed that 24 (47%) are currently linked to cardiogenesis or heart functions (Table S12). In this context, their identified putative interactions with heart developmental genes may indicate a specific role in cardiogenic regulation. More importantly, we suggest that the remaining 27 miRNAs should be considered as candidates for cardiac regulators. These include miR-139-3p targeting *Dicer1*, *Nedd4*, *Sox11*, and *Ednra*, as well as miR-540-3p targeting *Nrp2*, *Caxadr*, *Heg1*, and *Ttn* (Figure 7C).

HeartMir: An Interactive Resource for Identification of miRNA-Induced Gene Regulation during Heart Development and Maturation

We implemented a freely accessible database called HeartMiR, available at http://heartmir.sysbiolab.eu (Supplemental Information). It serves as a comprehensive tool for query, visualization, and identification of regulatory interactions of miRNAs with potential relevance in heart development and maturation. Its use is intuitive, following the scheme outlined in Figure 8. Thus, HeartMiR can be queried for all interactions of miRNAs and genes or for specific miRNA-mRNA interactions. Several gene or miRNA identifiers can be used to define a query. Currently, accepted gene identifiers include the gene name, gene symbol, Entrez Gene ID (using NCBI annotation), and its corresponding Affymetrix ID. Identifiers for miRNAs, which can be used for querying, include miRBase, Affymetrix, and Transcript IDs. Additionally, a threshold for correlation can be set, resulting in the exclusion of interactions with low absolute correlation between miRNAs and their corresponding target genes. Correlation of expression is measured using Kendall's tau coefficient, because we found the Pearson correlation to be too sensitive to large expression changes occurring between E19.5 and the young adult stage. The

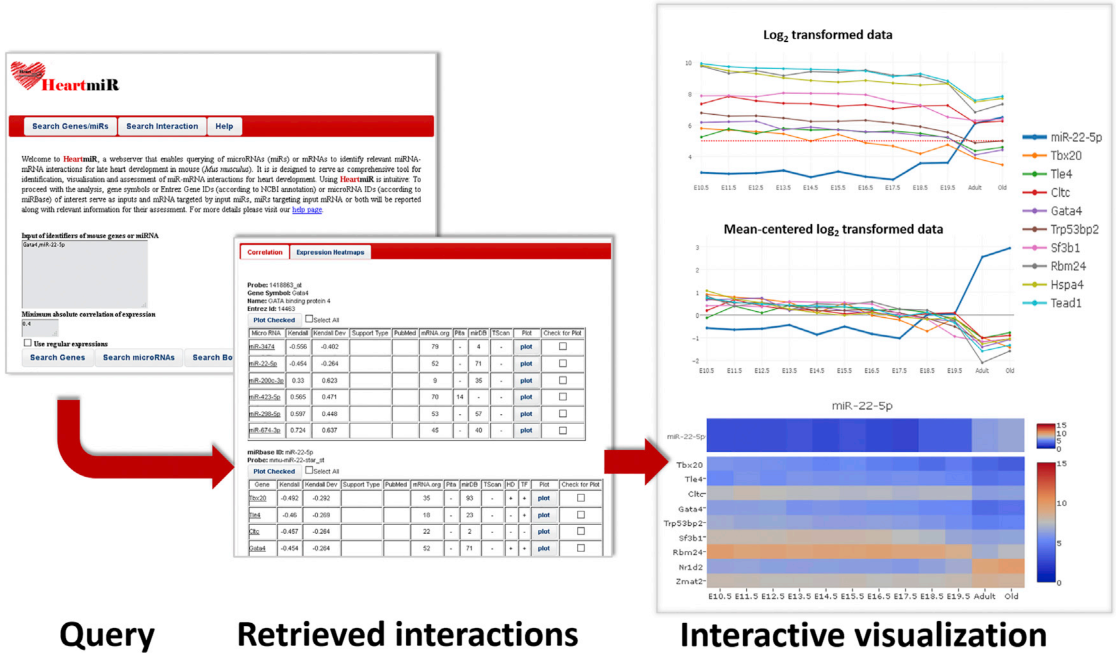

Figure 8. Workflow Scheme for HeartMir

Following input of miRNA or gene identifiers (here: *Gata4* and *mirR-22-5p*), HeartMir can be queried for putative miRNA-target gene interactions. All retrieved interactions are listed in tables, along with additional information. Subsequently, miRNA (*mirR-22-5p*) and target genes (*Tbx20* and *Gata4*) can be selected and their profiles visualized and interactively analyzed. Gene expression data are plotted as $\log_2$ intensities with or without mean centering. The former provides a more informative presentation of the absolute expression levels, whereas the latter shows expression changes of transcripts and their (anti-) correlation more clearly.

Kendall correlation can be used for the complete time series or with separate correlation coefficients for developmental time points only ("Kendall Dev"). This enables a more sensitive examination of how strongly the expression of miRNAs and their targets are (anti-) correlated during embryonic development (E10.5–E19.5).

The results of all queries are shown in tables. For each of the queried genes and miRNAs, a separate table displays these interactions, along with additional information. For experimentally derived interactions, the relevant PubMed references are given. To facilitate assessment of predicted interactions and a comparison of their scores, we ranked the scores and converted them to percentiles for each resource, separately. Thus, values between 1 and 100 are displayed for computationally predicted interactions used in HeartMiR. For instance, a value 1 signifies that the score of the interaction is within the top 1% of all scores for the corresponding resource; a value of 2 signifies that the score is in top 1%–2%. For gene targets, the result table also indicates whether they were assigned to processes related to heart development (GO: 0007507) or transcription regulation (GO: 0003700) in GO. These tables can be interactively sorted and filtered by increasing the threshold for absolute correlation. Finally, the expression profiles of miRNAs and target genes can be visualized as interactive line plots or heatmaps.

DISCUSSION

At present, there is no comprehensive compendium of heart-associated miRNAs, neither is there a clear understanding of how they contribute to the development of a fully mature and functional heart. In addition to providing fundamental biological knowledge, understanding their actions on heart development and function might provide crucial clues for regenerative treatment of heart diseases.[35] To facilitate such endeavors, we integrated known or predicted miRNA-mRNA interactions with expression data from this study. This resource is available as a user-friendly database (http://heartmir.sysbiolab.eu/). In order to reduce the transcripts noise from other cell types interfering with the heart developmental transcripts, we analyzed only transcripts that were up- or downregulated at least 4-fold at one stage of development.

Our findings suggest that the composition of cardiac ion channels undergoes major remodeling during development, as many of their components were differentially expressed during the various stages of heart development, compared with adult hearts. Among them, the expression levels of the *Cacna2d2* and *Cacna1h* declined, whereas the expression of *Cacna1g* showed reciprocal expression changes during heart development. Such remodeling may have physiological relevance, as *Cacna1g* and *Cacna1h* encode for the distinct subtypes of T-type Ca^{2+} channels: $Ca_v3.1$ (α1G) and $Ca_v3.2$ (α1H), which are crucial for electrical conduction in the atria.[36] Furthermore, it has been reported that disruption of *Cacna2d2* results in abnormalities in heart development.[37] The expression levels of potassium channels (*Kcna5* and *Kcnb1*) were very low during all developmental stages but were strongly upregulated in adult heart tissue. *Kcna5* encodes for the

voltage-gated K^+ channel (Kv1.5), which has emerged as a promising target for the treatment of atrial fibrillation.[38,39] The voltage-gated K^+ channel KCNB1 is mainly expressed in the heart, brain muscle, and pancreas[40] and is a key player in apoptotic programs associated with oxidative stress in the cardiovascular system.[41] With a transient maximum at E15.5, *Kcna4* displayed a distinctly different pattern that warrants further investigation, as its role during heart development has not yet been explored. A similar transient expression maximum was found for *Clca3a1* (*Clca1*), which encodes for a calcium-activated chloride channel contributing to the regulation of cellular volume. It has been speculated that *Clca3a1* is involved in arrhythmogenesis of the heart.[42]

An interesting by-product of our experimental design was the identification of genes that showed gender- or age-dependent expression. For instance, *ATP1A2* was found to be upregulated in old adult male compared with old adult female heart tissue. It belongs to the subfamily of Na^+/K^+-ATPase membrane proteins, regulating the electrochemical gradient in the cardiomyocytes and other cell types by transporting Na^+ and K^+ against their intracellular and extracellular concentrations. Similar upregulation was detected for *gelsolin*, *Gdf15*, members of the *Egr* and *Nr4a* protein families, and *Pah* in our study. Increased protein levels of *gelsolin* have previously been found in several organs of old rats.[43] In senescent human fibroblasts, an enhanced aging-associated resistance to apoptosis was observed for increased *gelsolin* expression. GDF15 is a cytokine having increased expression levels in heart degenerative diseases[44] and is induced under various stress conditions by the early growth response protein-1 (EGR-1), whose transcript increased in abundance over time in our study, together with those of its paralogs *Egr-2* and *Egr-3*. Members of the nuclear receptor subfamily 4, group A are transcriptional regulators of metabolism and energy balance that are induced by a pleiotropy of stimuli and processes. Recently, it has been hypothesized that they could serve as targets for anti-aging interventions.[45] Analysis of different microarray studies shows that aging led to consistently increased expression of phenylalanine hydroxylase (*Pah*) in murine hearts.[46] In summary, it appears that the male heart displays higher expression of known age-related marker genes compared with the female heart. We also identified genes that were downregulated in the male heart. These included *Myl7* (encodes for myosin light chain 2a) and *Myl4* (encodes for myosin light chain 1), two cardiac-specific cytoskeletal genes that are key regulators of heart contraction.

Of particular interest are genes encoding for lncRNAs, which are broadly defined as non-coding transcripts of length of 200 nt or more. Several lncRNAs are known to play key roles during heart development and have been associated with the regulation of gene expression through a variety of mechanisms on transcriptional, post-transcriptional, and epigenetic levels. In this context, a recent study identified a mouse-specific lncRNA termed Braveheart, expressed in embryonic stem cells and in mature heart tissue.[24] It was shown that Braveheart strongly enhances the cardiac commitment by acting as a decoy for SUZ12 (a member of the polycomb repressive complex 2 [PRC2]), thereby releasing MESP1 (a master regulator of cardiac differentiation) from PRC2 suppression. In this study, we found 25 lncRNAs with significant differential expression. Given the relevance of lncRNAs in gene regulation, this set of lncRNAs provides an attractive list of candidates for future study.

In addition to TFs and lncRNAs, miRNAs constitute another important class of regulators of gene expression. They have pivotal roles in developmental processes and are implicated in various diseases. Despite the relatively early association of miRNAs with heart-specific expression, a comprehensive capture of miRNA abundance in the developing heart has never been undertaken. The expression of miR17-5p was gradually downregulated from E10.5 to E19.5 and declined again in adult heart tissue (Figure 4). This supports a role in the regulation of proliferation of cardiac cells, as recently reported.[17] Expression in miR-29a-3p, miR-195a-5p, and miR-1a was relatively low at all developmental stages but was high in adult hearts. More recently, it has been shown that miR-29-3p is highly upregulated in adult hearts, as well as under pathological conditions, such as hypertrophic cardiomyopathy.[26,27] Expression patterns for miR-199a-3p and miR-133a showed maxima and minima at developmental stages E12.5 and E13.5, respectively. Expression of miR-199a-3p has been reported in the adult human heart and is correlated with heart failure.[28,29] In addition, miR199a-3p expression plays a pivotal role for cardiomyocytes survival.[30] The expression of miR-1 also was high in mature hearts compared with all embryonic stages. Notably, miR-1 belongs to the so called "miR combo" (including miR-133a) used to reprogram somatic cells *in vitro* and *in vivo*.[31–33] The expression of the major miRNA candidates obtained in our microarray study supports their involvement in heart development and cardiac tissue maintenance.[17,32,47–50] Among the miRNAs negatively correlated with heart development genes, we identified three miRNAs belonging to the let7 family (Table S11), which are upregulated during heart development. It has been previously reported that let7 family miRs are upregulated in the murine developing heart (E12.5, E14.5, E16.5, and E18.5; for review, see Bao et al.[51]).

Surprisingly, more upregulated than downregulated DEmiRs were detected during heart development. Assuming that the main regulatory activity of miRNA is post-transcriptional repression, this suggests that DEmiRs tend to function as suppressors of gene expression that is characteristic of mature tissue. This is a remarkable finding, as we can equally imagine the opposite scenario occurring, where DEmiRs are downregulated to enhance the expression of target genes that are characteristic for embryonic development. The consistent overrepresentation of upregulated DEmiRs indicates that the first mode of action is more common during heart development. The global analysis of the miRNA transcriptome also revealed a clear segregation between embryonic and mature tissue samples, indicating that miRNA expression in the developing heart is substantially different from the mature heart. Differences between the late stage of development (E19.5) and mature state define a framework for expression changes during postnatal maturation. Given that we detected a large variation between miRNA abundance in

embryonic and mature tissue, we expect that miRNAs contribute substantially to this maturation process. Indeed, a recent comparison of miRNA profiles of *in vitro* derived human cardiomyocytes identified a maturation-enhancing role for let-7g.[52] The inclusion of profiles of young and old heart tissue in our dataset enabled us to identify miRNAs that undergo characteristic postnatal changes and might drive the maturation process. We applied such template-based detection to the case of miR-17-5p, which has been linked to cardiomyocyte proliferation in neonatal and adult cardiomyocytes, to identify miRNAs that showed similar expression profiles. As shown in Figure S7, miR-122-5p and miR-20a-5p displayed a very similar expression pattern, with downregulation occurring in the mature heart. They are predicted to interact with two and six heart developmental genes, of which two (*Bmpr1a* and *Sox4*) and one (*Id1*) are also regulators of transcription, as indicated by GO annotation, respectively. The addition of transcriptome profiles for neonatal and postnatal heart tissue in future studies will help delineate the different profiles during maturation. In general, such template-based detection could be also applied to other expression patterns identified in this study.

The transcriptome study was carried out using a microarray platform, which has advantages but also limitations compared to RNA sequencing (RNA-seq) approach. The latter is a newer technology that is still relatively costly and requires a more complex and time-consuming process of storing and analyzing of the generated data.[53] Also, the transcriptome findings from RNA-seq can be very much depending on the parameter settings, specifically for low expressed genes. On the other hand, a major advantage of the RNA-seq is the identification of longer reads with less starting material.[54] Moreover, RNA-seq can identify previously unknown transcripts in contrast to the microarrays, which identify only transcripts for which probes have been included on the microarray.[55] Nevertheless, one of the assets of microarrays is the availability of numerous published datasets, which allows an easy reanalysis and comparison with newly generated data by other scientists.

Overall, the dual profiling of the poly(A)-RNA and miRNA transcriptome not only provided a comprehensive characterization of murine heart development but also promises to be an excellent basis to establish the regulatory molecular networks involved in heart development. Our study identified several miRNAs already reported to be associated with heart development across different studies,[48,49] as well as many others that appear to be important regulators but are yet to be explored.[56] In creating the open access database and interactive visualization tools implemented in HeartMiR, we provide a computational resource that will contribute to identification of miRNAs involved in regulating heart development.

MATERIALS AND METHODS

Tissue Collection and Treatment

Fetal hearts were isolated from E10.5 to E19.5 embryos dissected from OF1 pregnant mice. Young (10-week-old) and old (10-month-old) adult hearts of OF1 mice were also isolated as completely developed hearts to compare with embryonic stages. At each time point, three pups were collected as biological replicates in three 1.5-mL microtubes. Biological replicates included hearts from one female pup, one male pup, and one replicate with mixed male and female hearts per time point from E12.5 onward. Collection of heart material from young and old adult mice was carried out similarly. At time points E10.5 and E11.5, only mixed samples were used, because of incomplete sex differentiation. Disaggregation of heart tissue was performed by mechanical dissociation, using a tissue homogenization machine (Precellys24; Bertin Instruments, France). For tissue homogenization, 700 μL lysis buffer Trizol (QIAGEN, Hilden, Germany) was added to each tube and single-cell dissociation was performed twice, using the Precellys24 for 20 s at 500 rpm. These animal experiments were approved by the governmental animal care and use office (Landesamt für Natur, Umwelt und Verbraucherschutz Nordrhein-Westfalen, Recklinghausen, Germany; reference number 84-02.05.50.15.003).

Microarray Data Processing and Data Quality Assessment

Affymetrix Mouse Genome 430 2.0 arrays and Affymetrix miRNA 3.0 arrays were used to profile gene and miRNA expression during the mouse embryo cardiogenesis, respectively. RNA from homogenized heart tissue from different time points was isolated using miRNeasy mini kits (QIAGEN) with on-column DNase digestion following the manufacturer's instructions. Microarray labeling and hybridization techniques have been described in detail previously.[57]

Microarray Data Analysis

Microarray data were processed using the robust multi-array average (RMA) method on the R/Bioconductor platform. Variability between samples was visualized using PCA, hierarchical clustering, and density plots, implemented in R using the entire poly(A)-RNA and miRNA datasets. For differential expression analysis, the Bioconductor package *limma*[58] and multivariate empirical Bayes statistic were applied, enabling comparison of expression values between different time points.[59] To reduce noise in the data, an expression threshold was set. Genes or miRNA were only considered to be expressed and included for further analysis when the $\log_2$ intensity produced by RMA for the corresponding probe set was larger than five at one time point (at least). The 45101 probe sets on the Affymetrix Mouse Genome targeted 20,702 genes, with unique Entrez Gene IDs, of which 11,358 were found be expressed at one time point (at least). Samples from the 10-week-old adult hearts and from E19.5 were used as references to calculate differential expression. To evaluate gender-specific gene expression, samples from the same time points were paired and the overall contrast between male and female samples was calculated. This is analogous to a classical paired t test. Samples from E12.5 were excluded, as sex differentiation had not been concluded at this stage. Genes were defined as differentially expressed when the corresponding adjusted p value was lower than 10^{-5} and an absolute log2 fold change in expression of greater than 2 was recorded. To visualize expression profiles in Figures 2 and 3, the (logged) intensities produced by RMA were inverse $\log_2$ transformed.

The full mRNA and miRNA expression datasets have been deposited in the NCBI Gene Expression Omnibus (GEO GEO: GSE93271).

Clustering and Enrichment Analyses of Differentially Expressed Genes and miRNAs

To cluster DEGs and DEmiRs, the fuzzy c-means algorithm within the R/Bioconductor package *Mfuzz* was used.[60] Expression profiles of DEGs and DEmiRs were standardized, i.e., the mean value was set to zero and the SD was scaled to one for each DEG and DEmiR. The fuzzification parameter m was kept at its default value ($m = 2$). The number of clusters was selected based on the minimum distance between cluster centroids (as implemented in the *Dmin* function of the *Mfuzz* package), as well as inspection of the visualized cluster patterns. To facilitate interpretation, the cluster index was sorted based on the cluster profile, starting with downregulated clusters. GO enrichment analysis for these clusters was carried out in R using the Bioconductor packages org.Mm.eg.db[61] and GOstats.[62] To reduce the number of correlated categories detected as enriched, a hypergeometric test conditioned on the GO tree structure was applied.[62]

Integrative Analysis of Expression Data and Potential Regulatory Interactions

To obtain a comprehensive set of potential miRNA targets, we collated miRNA-mRNA interactions from five publically available resources: microRNA.org;[63] Pita;[64] miRDB;[65] TargetScan—providing computationally predicted interactions;[66] and MirTarBase,[67] which included interactions based on experimental evidence. To obtain interactions with high confidence, the computationally predicted interactions were filtered according to recommendations of the creators of these resources (see Supplemental Information for details). Interactions were discarded when miRs and mRNAs were found not to be expressed at any time point of the time series. Filtering of interactions resulted in the identification of 102,083 potential interactions between 386 miRNAs and 9,211 target genes. Correlation between mRNAs and miRNAs was calculated using the Kendall rank correlation, which provides a more robust measure of the similarity of expression profiles than a standard Pearson correlation.

Statistical Analysis

If not otherwise indicated in the text, the analysis was performed using a Bayesian moderated t test as implemented in the Bioconductor package limma and adjusted p values $< 10^{-5}$ were considered to be statistically significant.

SUPPLEMENTAL INFORMATION

Supplemental Information includes Supplemental Text, six figures, and twelve tables and can be found with this article online at https://doi.org/10.1016/j.omtn.2018.04.018.

AUTHOR CONTRIBUTIONS

D.S. isolated the heart tissue samples from the embryos. R.G.A.S. contributed to the isolation of the heart tissue. S.R. isolated the RNA and performed the poly-A RNA and miRNA Affymetrix analysis. R.S.R.M., M.E.F., and J.P.P. performed the bioinformatic analysis, contributed to the interpretation of the results, and constructed the HeartMiR database. A.S. supervised the study, interpreted the results, and wrote the manuscript in consultation with R.S.R.M. and M.E.F. R.S.R.M., M.E.F., J.P.P., J.H., D.S., and A.S. edited the manuscript.

CONFLICTS OF INTEREST

The authors declare no conflict of interest.

ACKNOWLEDGMENTS

This work was supported by the DFG (SA 568/17-2), the DAAD program (project ID: 57213647), and national Portuguese funding through the FCT (Fundação para a Ciência e Tecnologia) toward the ProRegeM PhD programme (PD/BD/105894; to R.S.R.M.), the bilateral FCT/DAAD collaboration 2016/2017, Research Centre grants (UID/BIM/04773/2013 – CBMR and UID/Multi/04326/2013 – CCMAR), a postdoc scholarship SFRH/BPD/96890/2013 (to J.P.P.), and developmental grant IF/00881/2013 (to M.E.F.).

REFERENCES

1. Srivastava, D. (2006). Making or breaking the heart: from lineage determination to morphogenesis. Cell *126*, 1037–1048.
2. Brand, T. (2003). Heart development: molecular insights into cardiac specification and early morphogenesis. Dev. Biol. *258*, 1–19.
3. Arceci, R.J., King, A.A., Simon, M.C., Orkin, S.H., and Wilson, D.B. (1993). Mouse GATA-4: a retinoic acid-inducible GATA-binding transcription factor expressed in endodermally derived tissues and heart. Mol. Cell. Biol. *13*, 2235–2246.
4. Bruneau, B.G., Nemer, G., Schmitt, J.P., Charron, F., Robitaille, L., Caron, S., Conner, D.A., Gessler, M., Nemer, M., Seidman, C.E., and Seidman, J.G. (2001). A murine model of Holt-Oram syndrome defines roles of the T-box transcription factor Tbx5 in cardiogenesis and disease. Cell *106*, 709–721.
5. Cai, C.L., Liang, X., Shi, Y., Chu, P.H., Pfaff, S.L., Chen, J., and Evans, S. (2003). Isl1 identifies a cardiac progenitor population that proliferates prior to differentiation and contributes a majority of cells to the heart. Dev. Cell *5*, 877–889.
6. Lin, Q., Schwarz, J., Bucana, C., and Olson, E.N. (1997). Control of mouse cardiac morphogenesis and myogenesis by transcription factor MEF2C. Science *276*, 1404–1407.
7. Lyons, I., Parsons, L.M., Hartley, L., Li, R., Andrews, J.E., Robb, L., and Harvey, R.P. (1995). Myogenic and morphogenetic defects in the heart tubes of murine embryos lacking the homeo box gene Nkx2-5. Genes Dev. *9*, 1654–1666.
8. Olson, E.N. (2006). Gene regulatory networks in the evolution and development of the heart. Science *313*, 1922–1927.
9. Meganathan, K., Sotiriadou, I., Natarajan, K., Hescheler, J., and Sachinidis, A. (2015). Signaling molecules, transcription growth factors and other regulators revealed from in-vivo and in-vitro models for the regulation of cardiac development. Int. J. Cardiol. *183*, 117–128.
10. Katz, M.G., Fargnoli, A.S., Kendle, A.P., Hajjar, R.J., and Bridges, C.R. (2016). The role of microRNAs in cardiac development and regenerative capacity. Am. J. Physiol. Heart Circ. Physiol. *310*, H528–H541.
11. Kwon, C., Han, Z., Olson, E.N., and Srivastava, D. (2005). MicroRNA1 influences cardiac differentiation in Drosophila and regulates Notch signaling. Proc. Natl. Acad. Sci. USA *102*, 18986–18991.
12. Stainier, D.Y., Lee, R.K., and Fishman, M.C. (1993). Cardiovascular development in the zebrafish. I. Myocardial fate map and heart tube formation. Development *119*, 31–40.
13. Zhao, Y., Ransom, J.F., Li, A., Vedantham, V., von Drehle, M., Muth, A.N., Tsuchihashi, T., McManus, M.T., Schwartz, R.J., and Srivastava, D. (2007). Dysregulation of cardiogenesis, cardiac conduction, and cell cycle in mice lacking miRNA-1-2. Cell *129*, 303–317.

14. Zhang, H.M., Kuang, S., Xiong, X., Gao, T., Liu, C., and Guo, A.Y. (2015). Transcription factor and microRNA co-regulatory loops: important regulatory motifs in biological processes and diseases. Brief. Bioinform. *16*, 45–58.

15. Bartel, D.P. (2004). MicroRNAs: genomics, biogenesis, mechanism, and function. Cell *116*, 281–297.

16. Zhao, Y., Samal, E., and Srivastava, D. (2005). Serum response factor regulates a muscle-specific microRNA that targets Hand2 during cardiogenesis. Nature *436*, 214–220.

17. Chen, J., Huang, Z.P., Seok, H.Y., Ding, J., Kataoka, M., Zhang, Z., Hu, X., Wang, G., Lin, Z., Wang, S., et al. (2013). mir-17-92 cluster is required for and sufficient to induce cardiomyocyte proliferation in postnatal and adult hearts. Circ. Res. *112*, 1557–1566.

18. Zhu, H. (2016). Forkhead box transcription factors in embryonic heart development and congenital heart disease. Life Sci. *144*, 194–201.

19. Sassoon, D.A., Garner, I., and Buckingham, M. (1988). Transcripts of alpha-cardiac and alpha-skeletal actins are early markers for myogenesis in the mouse embryo. Development *104*, 155–164.

20. England, J., and Loughna, S. (2013). Heavy and light roles: myosin in the morphogenesis of the heart. Cell. Mol. Life Sci. *70*, 1221–1239.

21. Kelly, R.D., and Cowley, S.M. (2013). The physiological roles of histone deacetylase (HDAC) 1 and 2: complex co-stars with multiple leading parts. Biochem. Soc. Trans. *41*, 741–749.

22. Ponnalagu, D., Gururaja Rao, S., Farber, J., Xin, W., Hussain, A.T., Shah, K., Tanda, S., Berryman, M., Edwards, J.C., and Singh, H. (2016). Molecular identity of cardiac mitochondrial chloride intracellular channel proteins. Mitochondrion *27*, 6–14.

23. Li, X., Martinez-Fernandez, A., Hartjes, K.A., Kocher, J.P., Olson, T.M., Terzic, A., and Nelson, T.J. (2014). Transcriptional atlas of cardiogenesis maps congenital heart disease interactome. Physiol. Genomics *46*, 482–495.

24. Klattenhoff, C.A., Scheuermann, J.C., Surface, L.E., Bradley, R.K., Fields, P.A., Steinhauser, M.L., Ding, H., Butty, V.L., Torrey, L., Haas, S., et al. (2013). Braveheart, a long noncoding RNA required for cardiovascular lineage commitment. Cell *152*, 570–583.

25. Koo, B.H., Coe, D.M., Dixon, L.J., Somerville, R.P., Nelson, C.M., Wang, L.W., Young, M.E., Lindner, D.J., and Apte, S.S. (2010). ADAMTS9 is a cell-autonomously acting, anti-angiogenic metalloprotease expressed by microvascular endothelial cells. Am. J. Pathol. *176*, 1494–1504.

26. Fang, L., Ellims, A.H., Moore, X.L., White, D.A., Taylor, A.J., Chin-Dusting, J., and Dart, A.M. (2015). Circulating microRNAs as biomarkers for diffuse myocardial fibrosis in patients with hypertrophic cardiomyopathy. J. Transl. Med. *13*, 314.

27. Li, M., Wang, N., Zhang, J., He, H.P., Gong, H.Q., Zhang, R., Song, T.F., Zhang, L.N., Guo, Z.X., Cao, D.S., and Zhang, T.C. (2016). MicroRNA-29a-3p attenuates ET-1-induced hypertrophic responses in H9c2 cardiomyocytes. Gene *585*, 44–50.

28. Ovchinnikova, E.S., Schmitter, D., Vegter, E.L., Ter Maaten, J.M., Valente, M.A., Liu, L.C., van der Harst, P., Pinto, Y.M., de Boer, R.A., Meyer, S., et al. (2016). Signature of circulating microRNAs in patients with acute heart failure. Eur. J. Heart Fail. *18*, 414–423.

29. Vegter, E.L., Schmitter, D., Hagemeijer, Y., Ovchinnikova, E.S., van der Harst, P., Teerlink, J.R., O'Connor, C.M., Metra, M., Davison, B.A., Bloomfield, D., et al. (2016). Use of biomarkers to establish potential role and function of circulating microRNAs in acute heart failure. Int. J. Cardiol. *224*, 231–239.

30. Park, K.M., Teoh, J.P., Wang, Y., Broskova, Z., Bayoumi, A.S., Tang, Y., Su, H., Weintraub, N.L., and Kim, I.M. (2016). Carvedilol-responsive microRNAs, miR-199a-3p and -214 protect cardiomyocytes from simulated ischemia-reperfusion injury. Am. J. Physiol. Heart Circ. Physiol. *311*, H371–H383.

31. Jayawardena, T., Mirotsou, M., and Dzau, V.J. (2014). Direct reprogramming of cardiac fibroblasts to cardiomyocytes using microRNAs. Methods Mol. Biol. *1150*, 263–272.

32. Jayawardena, T.M., Egemnazarov, B., Finch, E.A., Zhang, L., Payne, J.A., Pandya, K., Zhang, Z., Rosenberg, P., Mirotsou, M., and Dzau, V.J. (2012). MicroRNA-mediated in vitro and in vivo direct reprogramming of cardiac fibroblasts to cardiomyocytes. Circ. Res. *110*, 1465–1473.

33. Jayawardena, T.M., Finch, E.A., Zhang, L., Zhang, H., Hodgkinson, C.P., Pratt, R.E., Rosenberg, P.B., Mirotsou, M., and Dzau, V.J. (2015). MicroRNA induced cardiac reprogramming in vivo: evidence for mature cardiac myocytes and improved cardiac function. Circ. Res. *116*, 418–424.

34. Futschik, M.E., and Carlisle, B. (2005). Noise-robust soft clustering of gene expression time-course data. J. Bioinform. Comput. Biol. *3*, 965–988.

35. Yan, S., and Jiao, K. (2016). Functions of miRNAs during mammalian heart development. Int. J. Mol. Sci. *17*, E789.

36. Curran, J., Musa, H., Kline, C.F., Makara, M.A., Little, S.C., Higgins, J.D., Hund, T.J., Band, H., and Mohler, P.J. (2015). Eps15 homology domain-containing protein 3 regulates cardiac T-type Ca2+ channel targeting and function in the atria. J. Biol. Chem. *290*, 12210–12221.

37. Ivanov, S.V., Ward, J.M., Tessarollo, L., McAreavey, D., Sachdev, V., Fananapazir, L., Banks, M.K., Morris, N., Djurickovic, D., Devor-Henneman, D.E., et al. (2004). Cerebellar ataxia, seizures, premature death, and cardiac abnormalities in mice with targeted disruption of the Cacna2d2 gene. Am. J. Pathol. *165*, 1007–1018.

38. Olson, T.M., Alekseev, A.E., Liu, X.K., Park, S., Zingman, L.V., Bienengraeber, M., Sattiraju, S., Ballew, J.D., Jahangir, A., and Terzic, A. (2006). Kv1.5 channelopathy due to KCNA5 loss-of-function mutation causes human atrial fibrillation. Hum. Mol. Genet. *15*, 2185–2191.

39. Schumacher-Bass, S.M., Vesely, E.D., Zhang, L., Ryland, K.E., McEwen, D.P., Chan, P.J., Frasier, C.R., McIntyre, J.C., Shaw, R.M., and Martens, J.R. (2014). Role for myosin-V motor proteins in the selective delivery of Kv channel isoforms to the membrane surface of cardiac myocytes. Circ. Res. *114*, 982–992.

40. Sesti, F., Wu, X., and Liu, S. (2014). Oxidation of KCNB1 K(+) channels in central nervous system and beyond. World J. Biol. Chem. *5*, 85–92.

41. Roder, K., and Koren, G. (2006). The K+ channel gene, Kcnb1: genomic structure and characterization of its 5′-regulatory region as part of an overlapping gene group. Biol. Chem. *387*, 1237–1246.

42. Duan, D.Y., Liu, L.L., Bozeat, N., Huang, Z.M., Xiang, S.Y., Wang, G.L., Ye, L., and Hume, J.R. (2005). Functional role of anion channels in cardiac diseases. Acta Pharmacol. Sin. *26*, 265–278.

43. Ahn, J.S., Jang, I.S., Kim, D.I., Cho, K.A., Park, Y.H., Kim, K., Kwak, C.S., and Chul Park, S. (2003). Aging-associated increase of gelsolin for apoptosis resistance. Biochem. Biophys. Res. Commun. *312*, 1335–1341.

44. Adela, R., and Banerjee, S.K. (2015). GDF-15 as a target and biomarker for diabetes and cardiovascular diseases: a translational prospective. J. Diabetes Res. *2015*, 490842.

45. Paillasse, M.R., and de Medina, P. (2015). The NR4A nuclear receptors as potential targets for anti-aging interventions. Med. Hypotheses *84*, 135–140.

46. Swindell, W.R. (2009). Genes and gene expression modules associated with caloric restriction and aging in the laboratory mouse. BMC Genomics *10*, 585.

47. Dimmeler, S. (2014). MicroRNAs in cardiovascular diseases and aging. FEBS J. *281*, 23.

48. Hodgkinson, C.P., Kang, M.H., Dal-Pra, S., Mirotsou, M., and Dzau, V.J. (2015). MicroRNAs and cardiac regeneration. Circ. Res. *116*, 1700–1711.

49. Thum, T., Catalucci, D., and Bauersachs, J. (2008). MicroRNAs: novel regulators in cardiac development and disease. Cardiovasc. Res. *79*, 562–570.

50. Zhao, J., Feng, Y., Yan, H., Chen, Y., Wang, J., Chua, B., Stuart, C., and Yin, D. (2014). β-arrestin2/miR-155/GSK3β regulates transition of 5′-azacytizine-induced Sca-1-positive cells to cardiomyocytes. J. Cell. Mol. Med. *18*, 1562–1570.

51. Bao, M.H., Feng, X., Zhang, Y.W., Lou, X.Y., Cheng, Y., and Zhou, H.H. (2013). Let-7 in cardiovascular diseases, heart development and cardiovascular differentiation from stem cells. Int. J. Mol. Sci. *14*, 23086–23102.

52. Kumarswamy, R., and Thum, T. (2013). Non-coding RNAs in cardiac remodeling and heart failure. Circ. Res. *113*, 676–689.

53. Zhao, S., Fung-Leung, W.P., Bittner, A., Ngo, K., and Liu, X. (2014). Comparison of RNA-seq and microarray in transcriptome profiling of activated T cells. PLoS ONE *9*, e78644.

54. Conesa, A., Madrigal, P., Tarazona, S., Gomez-Cabrero, D., Cervera, A., McPherson, A., Szcześniak, M.W., Gaffney, D.J., Elo, L.L., Zhang, X., and Mortazavi, A. (2016). A survey of best practices for RNA-seq data analysis. Genome Biol. *17*, 13.

55. Hunt, E.A., Broyles, D., Head, T., and Deo, S.K. (2015). MicroRNA detection: current technology and research strategies. Annu. Rev. Anal. Chem. (Palo Alto, Calif.) *8*, 217–237.

56. Ding, W., Li, J., Singh, J., Alif, R., Vazquez-Padron, R.I., Gomes, S.A., Hare, J.M., and Shehadeh, L.A. (2015). miR-30e targets IGF2-regulated osteogenesis in bone marrow-derived mesenchymal stem cells, aortic smooth muscle cells, and ApoE-/- mice. Cardiovasc. Res. *106*, 131–142.

57. Meganathan, K., Jagtap, S., Srinivasan, S.P., Wagh, V., Hescheler, J., Hengstler, J., Leist, M., and Sachinidis, A. (2015). Neuronal developmental gene and miRNA signatures induced by histone deacetylase inhibitors in human embryonic stem cells. Cell Death Dis. *6*, e1756.

58. Ritchie, M.E., Phipson, B., Wu, D., Hu, Y., Law, C.W., Shi, W., and Smyth, G.K. (2015). limma powers differential expression analyses for RNA-sequencing and microarray studies. Nucleic Acids Res. *43*, e47.

59. Tai, Y.C., and Speed, T.P. (2006). A multivariate empirical Bayes statistic for replicated microarray time course data. Ann. Stat. *34*, 2387–2412.

60. Kumar, L., and E Futschik, M. (2007). Mfuzz: a software package for soft clustering of microarray data. Bioinformation *2*, 5–7.

61. Carlson, M. (2014). org.Mm.eg.db: genome wide annotation for mouse. https://bioconductor.org/packages/release/data/annotation/html/org.Mm.eg.db.html.

62. Falcon, S., and Gentleman, R. (2007). Using GOstats to test gene lists for GO term association. Bioinformatics *23*, 257–258.

63. Betel, D., Wilson, M., Gabow, A., Marks, D.S., and Sander, C. (2008). The microRNA.org resource: targets and expression. Nucleic Acids Res. *36*, D149–D153.

64. Kertesz, M., Iovino, N., Unnerstall, U., Gaul, U., and Segal, E. (2007). The role of site accessibility in microRNA target recognition. Nat. Genet. *39*, 1278–1284.

65. Wong, N., and Wang, X. (2015). miRDB: an online resource for microRNA target prediction and functional annotations. Nucleic Acids Res. *43*, D146–D152.

66. Agarwal, V., Bell, G.W., Nam, J.W., and Bartel, D.P. (2015). Predicting effective microRNA target sites in mammalian mRNAs. eLife *4*, e05005.

67. Chou, C.H., Chang, N.W., Shrestha, S., Hsu, S.D., Lin, Y.L., Lee, W.H., Yang, C.D., Hong, H.C., Wei, T.Y., Tu, S.J., et al. (2016). miRTarBase 2016: updates to the experimentally validated miRNA-target interactions database. Nucleic Acids Res. *44* (D1), D239–D247.

OMTN, Volume *12*

Supplemental Information

Parallel Genome-wide Profiling of Coding and Non-coding RNAs to Identify Novel Regulatory Elements in Embryonic and Maturated Heart

Davood Sabour, Rui S.R. Machado, José P. Pinto, Susan Rohani, Raja G.A. Sahito, Jürgen Hescheler, Matthias E. Futschik, and Agapios Sachinidis

Supplementary Figures

Poly(A)-RNA

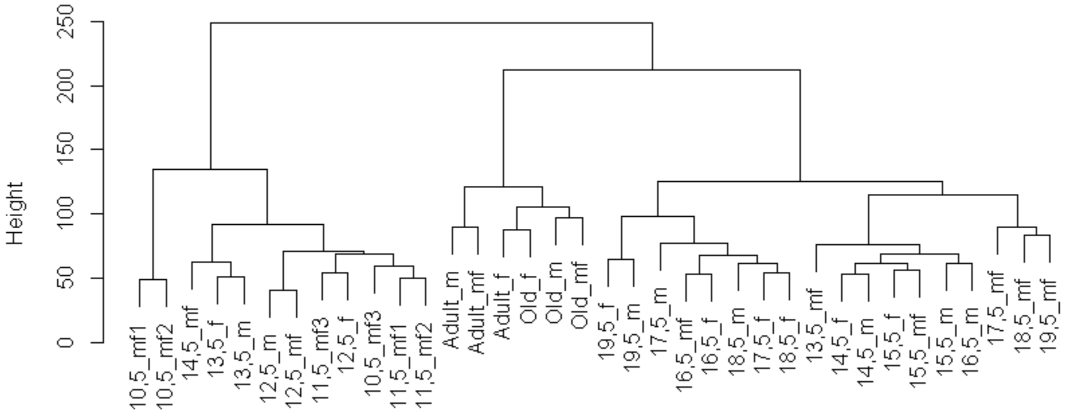

miRNA

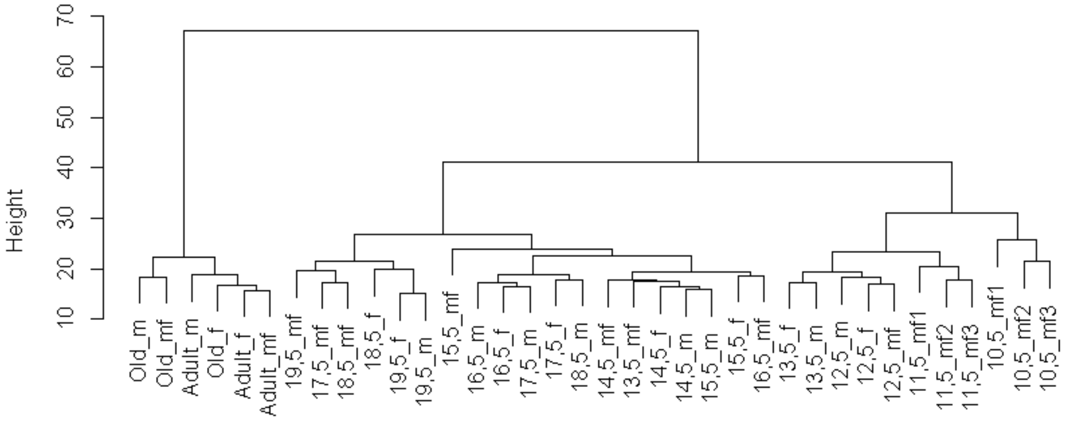

Figure S1. **Clustering dendrograms** produced based on both full poly(A)-RNA and miRNA microarray profiles.

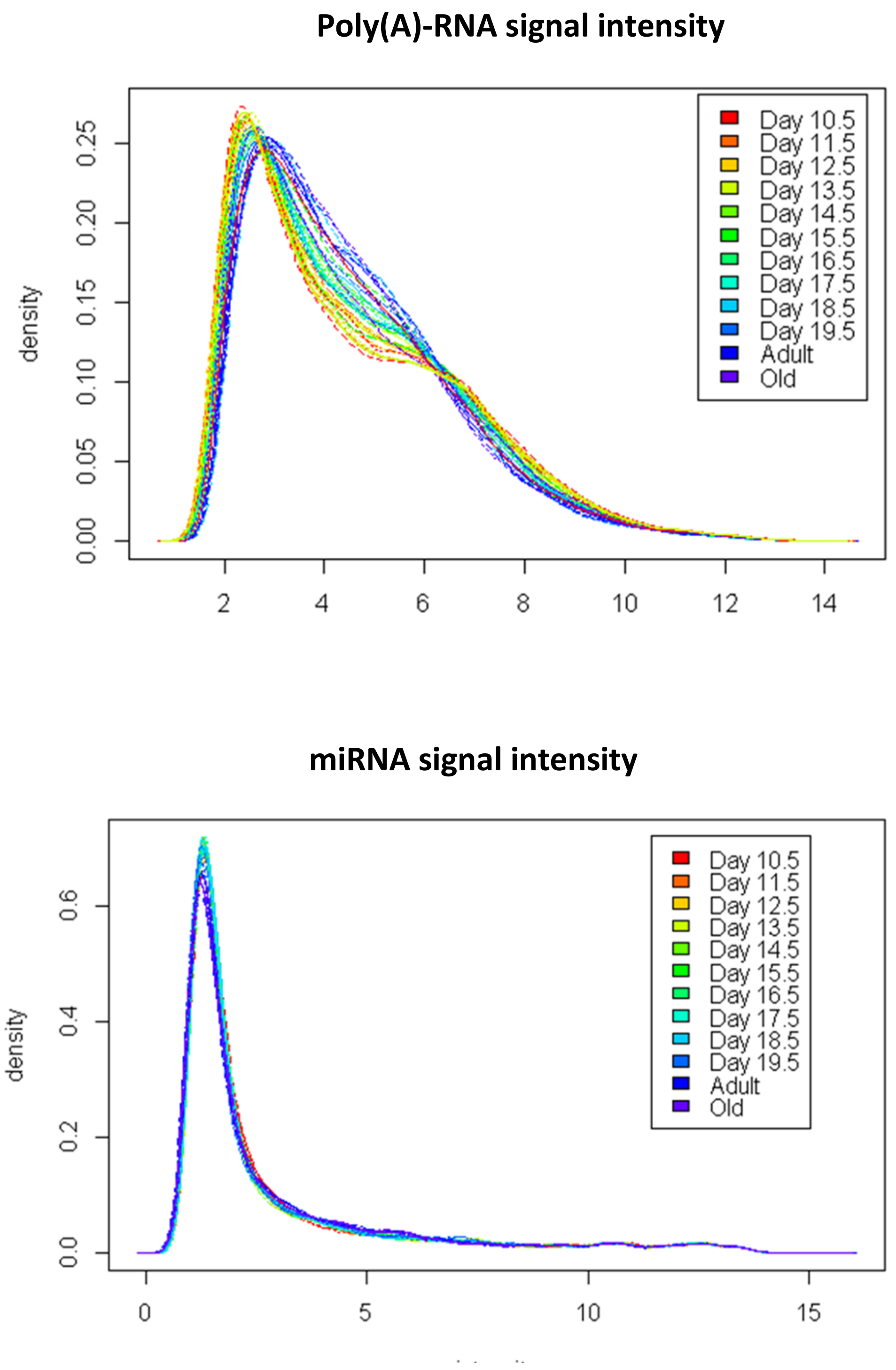

Figure S2. **Density plots of log2-tranformed expression intensities**. The distribution for poly(A)-RNA displays a notable shoulder for logged expression values around 6 at earlier developmental stages (E11.5, E12.5, E13.5).

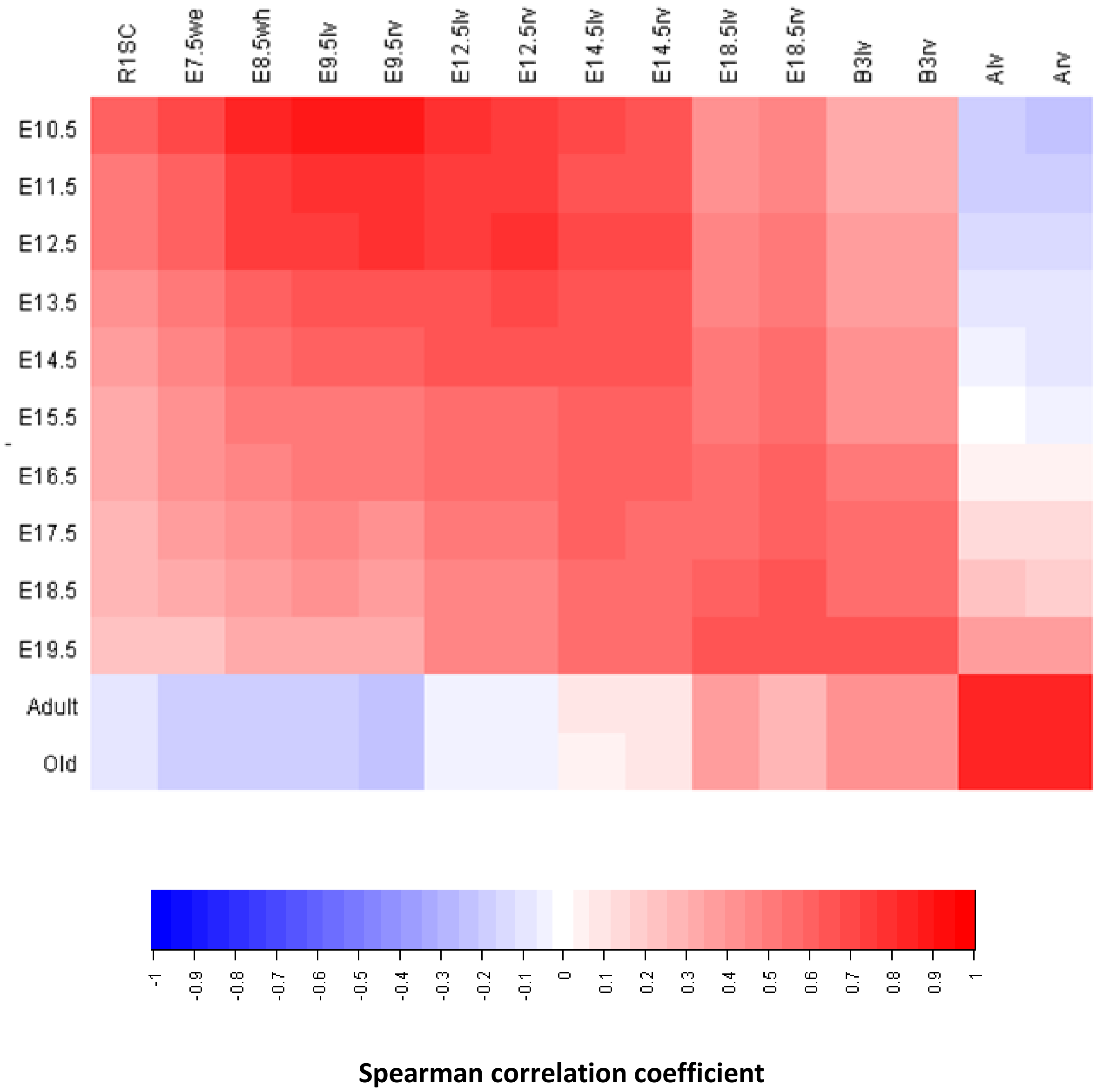

Figure S3. Heatmap of correlation coefficients derived by cross-correlation with alternative microarray study (Li et al., 2014). Color represent Spearman correlation coefficient between expression profiles from our study and the study by Li *et. al*. The bar below the heatmap displays the color scheme: red indicates positive correlation while blue indicates negative correlation (or anti-correlation). In the labels for the time series by Li *et. al.* "we" stands for "whole embryo", "wh" for "whole heart", "lv" for "left ventricle", "rv" for "right ventricle", and "B" day after birth. Moreover, this dataset included an expression profile of an undifferentiated murine embryonic stem cell line (R1SC).

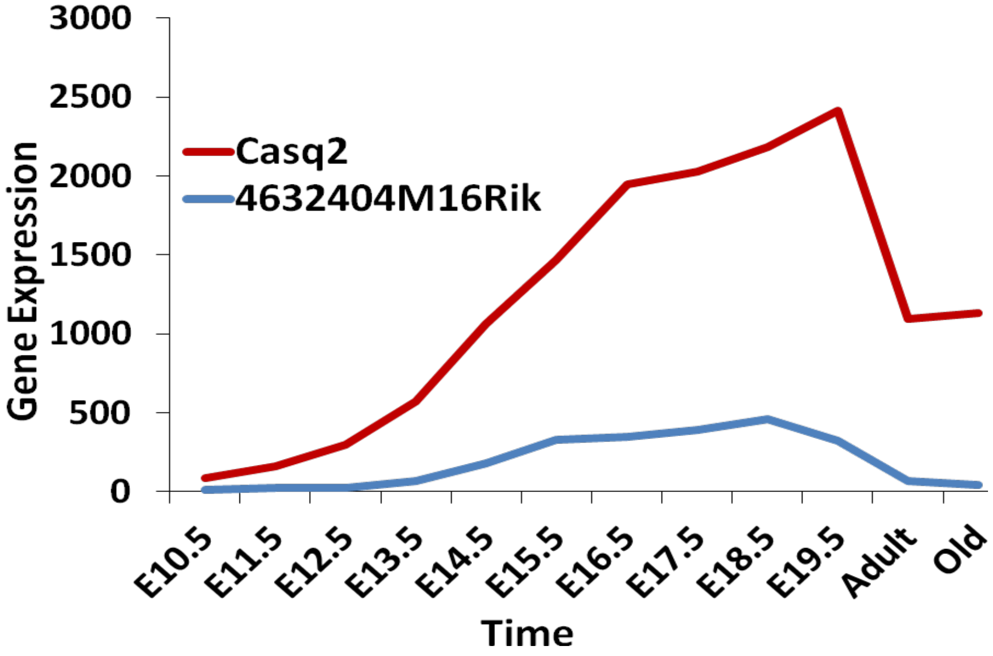

Figure S4: Expression profiles of Casq2 and the intronic lncRNA 4632404M16Rik. After reaching a peak at day 18.5, the expression of the lncRNA drops by a factor of ten in old tissue.

A

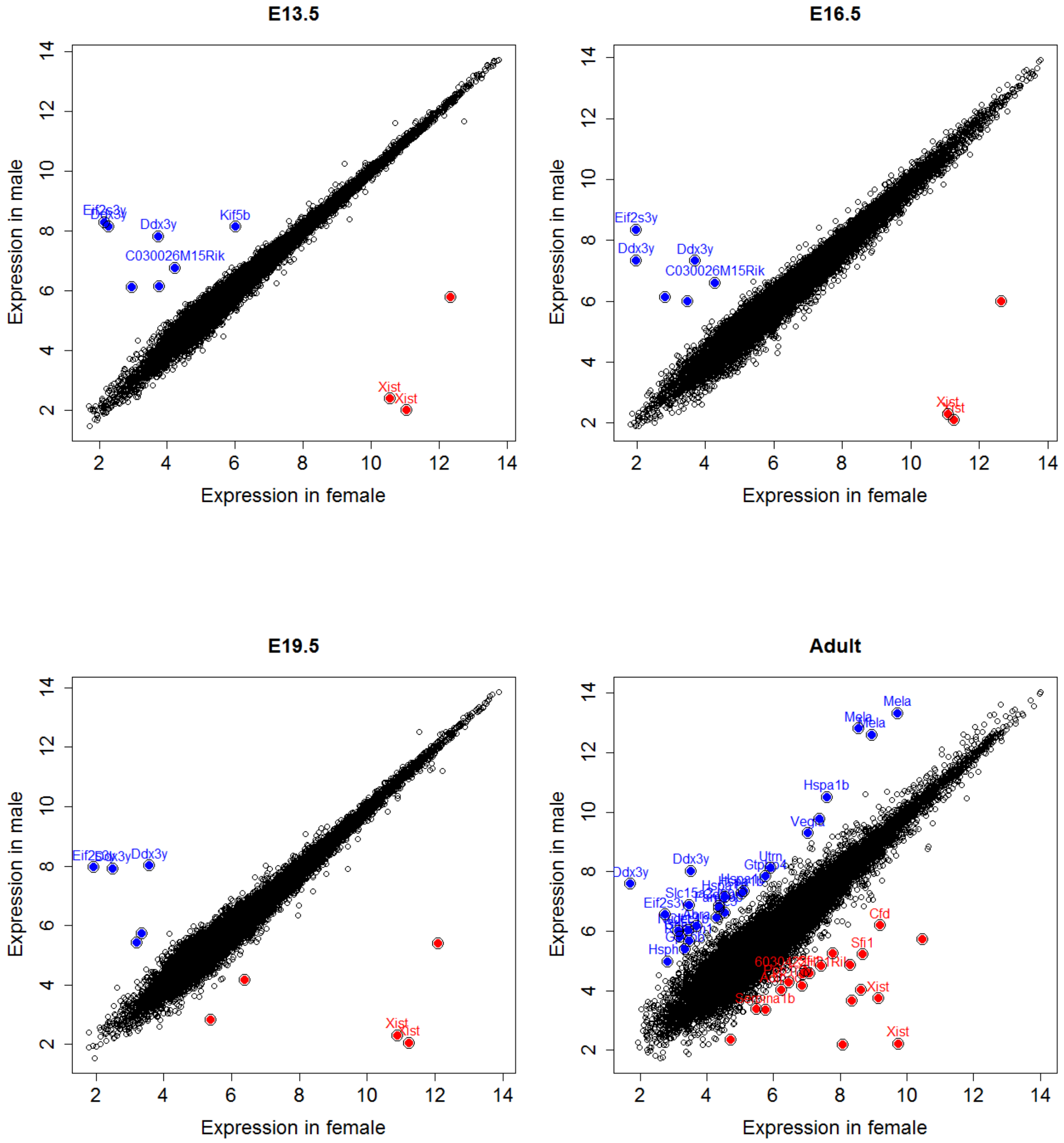
E13.5
Expression in male
Expression in female
Eif2s3y
Ddx3y
Ddx3y
Kif5b
C030026M15Rik
Xist
Xist
E16.5
Expression in male
Expression in female
Eif2s3y
Ddx3y
Ddx3y
C030026M15Rik
Xist
E19.5
Expression in male
Expression in female
Ddx3y
Xist
Adult
Expression in male
Expression in female
Mela
Mela
Mela
Hspa1b
Vegfa
Ddx3y
Ddx3y
Utrn
Slc15a2
Eif2s3y
Cfd
Sfi1
Xist
Xist

B

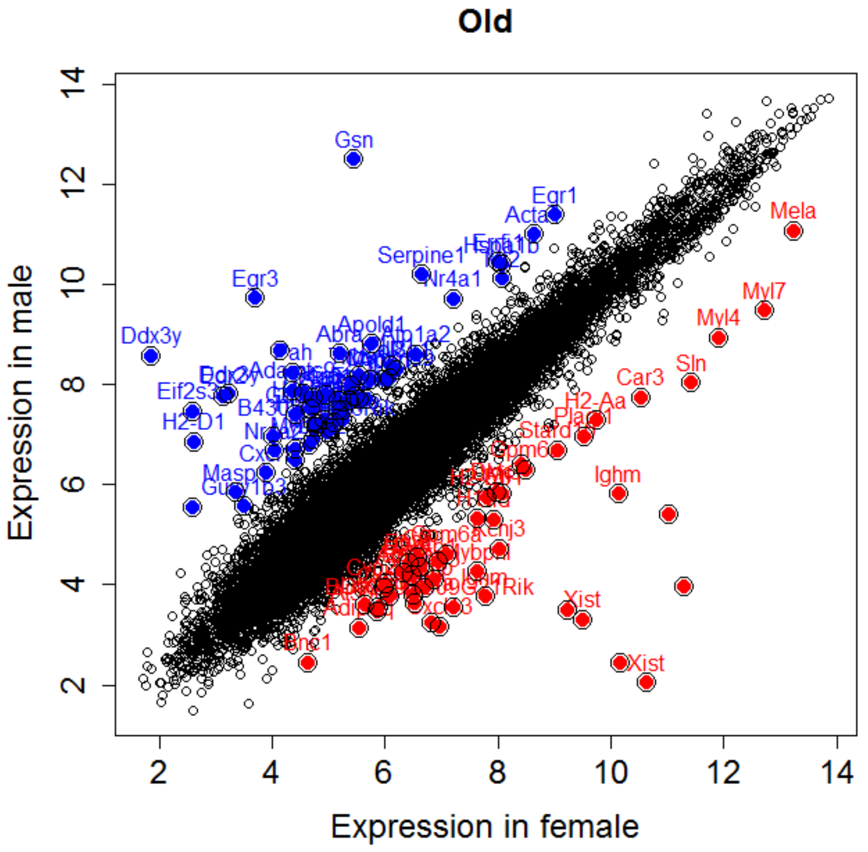

C

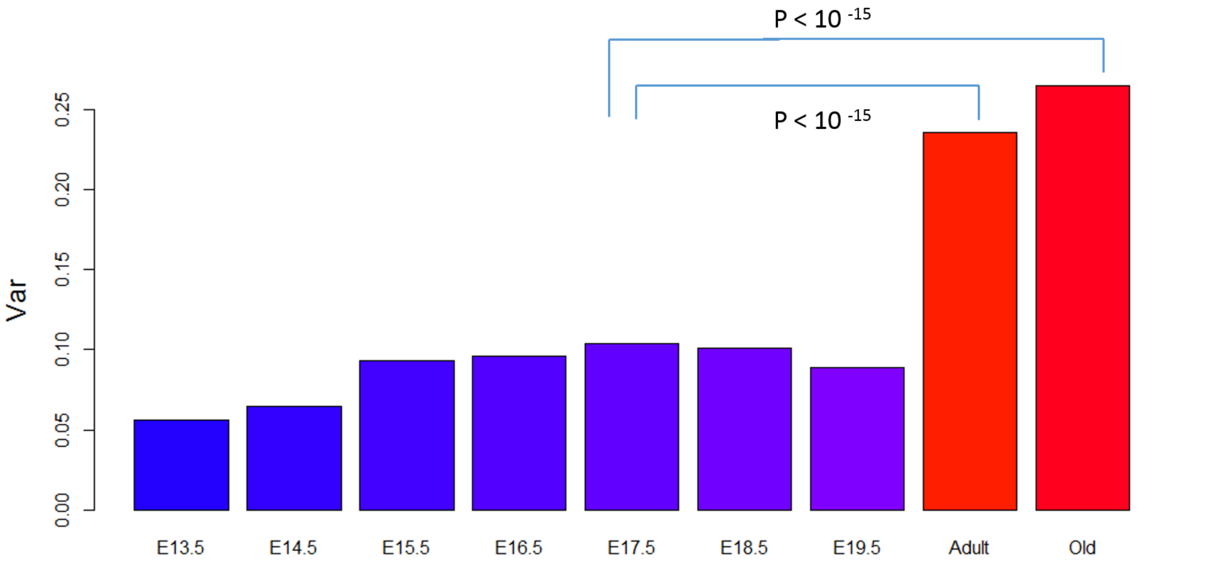

Figure S5. Comparison of gene expression in male and female samples. A) Scatterplots of logged signal intensities for male vs female samples of E13.5, E16.5, E19.5 and young adult mice. Red dots mark probe sets with signal intensities that are more than 4-fold larger in female than in male samples. Blue dots indicate probe sets with at least 4-fold larger expression in male samples. **B)** Scatterplot for old heart tissue. **C)** Variance of differential expression between male and female samples for different time points. P-values were derived using the F-test.

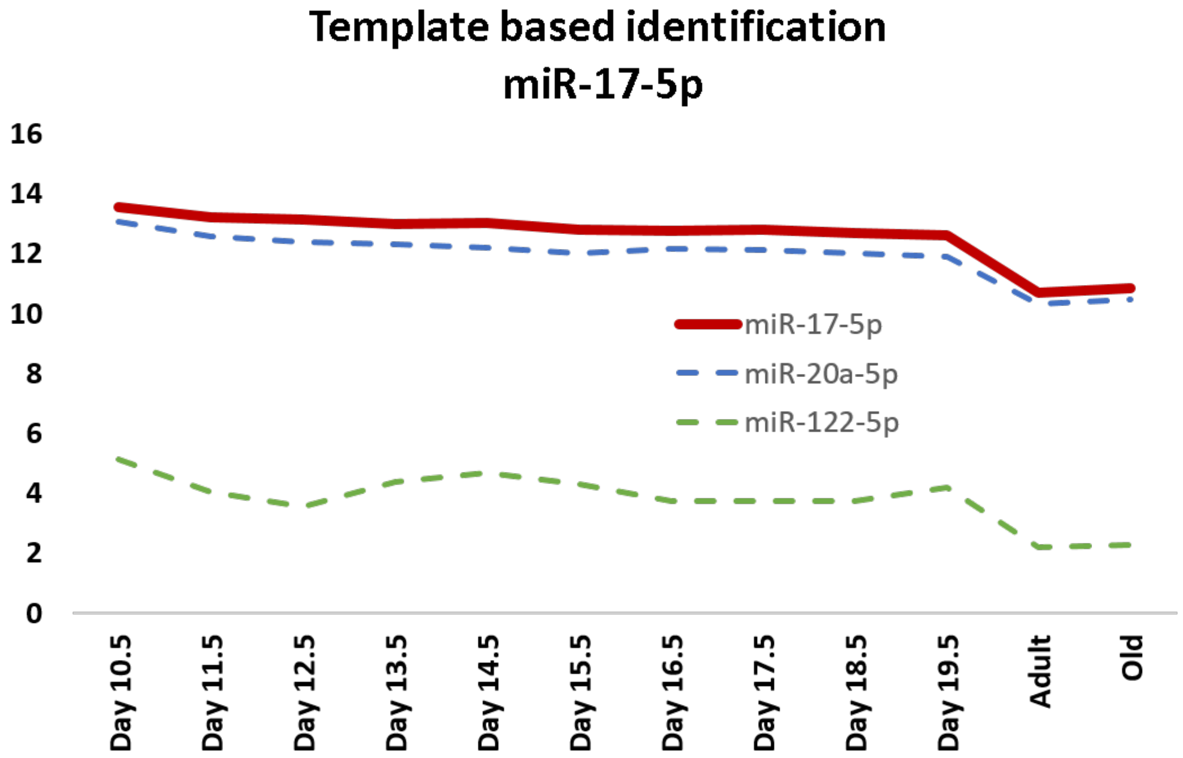

Figure S6. Example for detection of miRNAs with similar profiles. Here we used mir-17-5p as template and searched for miRNA with similar expression patterns in our miR transcriptome data set.

Supplementary Text

a. Comparison with previous microarray study of murine heart development

Recently, a similar study poly(A)-RNA abundance during the murine heart development was performed using the same type of the Affymetrix microarrays as we used in our study (Mouse Genome 430 2.0 arrays).[1] To assess the reproducibility of our study, we compared the expression values detected by the two studies.

For this purpose, we calculate the cross-correlation for the expression profiles captured at the different heart developmental stages. Such approach avoids the necessity that both studies include the same time points, which is not the case here. The alternative study of Li and co-workers[1] had lower temporal resolution (i.e. no measurements were taken for E10.5, E11.5, E13.5, E16.5, E17.5, E19.5), and started earlier during development making a direct alignment challenging. Furthermore, it included distinct tissue samples for left and right ventricle. Nevertheless, we would expect a high pair-wise correlation of similar time points using the cross-correlation approach if expression profiles of both studies agree. The expression profiles were defined here as the set of intensity values (averaged over replicates) of the 3540 Affymetrix probe sets that showed differential expression in our study. The microarray data by Li et al (2014)[1] were downloaded from GEO and processed in the same manner as ours. The results of the cross-correlation were displayed as heatmap, in which red colour represents correlation and the blue colour represents anti-correlation (**Figure S3**).

The comparison showed that the expression profiles for similar time points are highly correlated between the two studies. For example, a correlation of 0.87 was obtained for the expression values of E10.5 in our study and E9.5 of the alternative study. Inspection also reveals that both time series agree in their relative temporal alignment. The level of correlation decreases with increased distance in time. For instance, the measurements for adult tissues in both studies are strongly correlated, while anti-correlated with early developmental time points of the other study. Based on the obtained results we conclude that detected gene expression intensities in our study are consistent with those previously reported.[1]

b. Implementation of HeartmiR tool

HeartmiR is based on the integration of a large number of potential miRNAs-mRNAs interactions derived from different sources with the expression data that we obtained by parallel genome-wide profiling of mRNA and miRNA undertaken in our study. To obtain a comprehensive set of miRNA-mRNA interactions, we integrated five different datasets of miRNA-mRNA interactions that are publically available. These interactions are based on experiments[2] or computational predictions.[3-6] In order to obtain interactions of high confidence, each dataset was individually examined and filtered following the recommendations of the creators of the resources:

- **microRNA.org** (www.microrna.org): The two files labelled with "Good mirSVR score, Conserved miRNA" and the "Good mirSVR score, Non-conserved miRNA" were downloaded from www.microrna.org/microrna/ getDownloads.do. Interactions were subsequently merged into a single list and duplicated interactions were removed. To increase confidence, we eliminated all interactions that had a mirSVR score higher than -0.1 following the recommendation given by Betel.[7] This

produced a list of 92141 miRNA-RNA interactions.

- **Pita:** Mouse miRNA targets were obtained from the Download Page (https://genie.weizmann.ac.il/pubs/mir07/catalogs/PITA_sites_mm9_0_0_ALL.tab.gz). A minimal score of -10 was set according to the authors' indications in the FAQ/Notes page (https://genie.weizmann.ac.il/pubs/mir07/index.html). Applying this threshold, we obtained 19615 miRNA-mRNA interactions.
- **miRDB:** Predicted interactions were retrieved from the miRDB webportal (http://www.mirdb.org/miRDB/download/miRDB_v5.0_prediction_result.txt.gz). Following the recommendations stated on the Download page, a gene with a score higher that 50 should be considered as a likely miRNA target. Considering only interaction with a score higher than 50 resulted in 68035 miRNA-mRNA interactions.
- **TargetScan***:* Murine miRNA-mRNA interactions were downloaded from the TargetScan webpage (http://www.targetscan.org/mmu_71/). Only interactions with "Cumulative weighted context++ score" lower than -0.1 were to increase prediction confidence as suggested by Agarwal *et al.* (2015) resulting in 18612 interactions.
- **MirTarBase:** miRNA-mRNA interactions for mouse based on experimental data were downloaded from MirTarBase Version 6.1 (http://mirtarbase.mbc.nctu.edu.tw/cache/download/6.1/mmu_MTI.xls). The complete set of 13027 miRNA-mRNA interactions of the MirTarBase was included in HeartmiR.

For integration with the expression data, miRNA targets were mapped to their gene symbol and Entrez Gene ID. We only kept interactions for which the corresponding mRNAs and miRNAs have higher expression than 32 units for at least one time point. To facilitate assessment of predicted interactions and comparison of their scores, we ranked the scores and converted them to percentiles for each resource separately. This information is displayed when using HeartmiR.

c. Use of HeartmiR

HeartmiR can be queried for interactions with given miRNA and genes or for specific miRNA-mRNA interactions. Several gene or miRNA identifiers can be used to define the query. Currently, accepted gene identifiers include the gene name, gene symbol, Entrez Gene ID (according to NCBI annotation) and the corresponding Affymetrix ID. Identifiers for miRNAs that can used for querying include the miRBase ID, the Affymetrix ID and the Transcript ID. Additionally, a threshold for correlation can be set resulting in the exclusion of interactions with lower absolute correlation between miRNA and the corresponding target mRNA. For instance, setting a threshold of 0.5, only miRNA-mRNA interactions that have a Kendall correlation higher than 0.5 or lower than -0.5 will be showed. By default, the correlation threshold is set to 0.4.

The results of the query are shown in tables. For each of the queried genes and miRNAs, a separate table displays the interactions with additional information. For experimentally derived interactions, the relevant PubMed references are given. For computationally predicted interactions, a value between 1 and 100 is shown. This represents the percentile of the ranked score. The value 1 signifies that the score of the interaction is within the top 1% of all scores for the corresponding resource; a value of 2 signifies that the score is in top 1-2% and so on. Besides the Kendall correlation for the complete time

series, separate correlation coefficient for developmental time points only ("Kendall Dev") are shown. This can enable a better examination how strongly the expression of miRNAs and their targets are (anti-)correlated during the developmental phase (E10.5-E19.5). For gene targets, the table also indicates whether they were assigned to processes related to heart development (GO:0007507) or transcription regulation (GO:0003700) in Gene Ontology. The tables can be interactively sorted and further filtered by increasing the threshold for absolute correlation. Finally, the expression profiles of miRNAs and target genes can be visualised as interactive line plots or heatmaps.

References

1. Li, X, Martinez-Fernandez, A, Hartjes, KA, Kocher, JP, Olson, TM, Terzic, A*, et al.* (2014). Transcriptional atlas of cardiogenesis maps congenital heart disease interactome. *Physiol Genomics* **46**: 482-495.
2. Chou, CH, Chang, NW, Shrestha, S, Hsu, SD, Lin, YL, Lee, WH*, et al.* (2016). miRTarBase 2016: updates to the experimentally validated miRNA-target interactions database. *Nucleic Acids Res* **44**: D239-247.
3. Agarwal, V, Bell, GW, Nam, JW, and Bartel, DP (2015). Predicting effective microRNA target sites in mammalian mRNAs. *Elife* **4**.
4. Bartel, DP (2004). MicroRNAs: genomics, biogenesis, mechanism, and function. *Cell* **116**: 281-297.
5. Kertesz, M, Iovino, N, Unnerstall, U, Gaul, U, and Segal, E (2007). The role of site accessibility in microRNA target recognition. *Nat Genet* **39**: 1278-1284.
6. Wong, N, and Wang, X (2015). miRDB: an online resource for microRNA target prediction and functional annotations. *Nucleic Acids Res* **43**: D146-152.
7. Betel, D, Wilson, M, Gabow, A, Marks, DS, and Sander, C (2008). The microRNA.org resource: targets and expression. *Nucleic Acids Res* **36**: D149-153.
